# Isolation and characterisation of ΦcrAss002, a crAss-like phage from the human gut that infects *Bacteroides xylanisolvens*

**DOI:** 10.1186/s40168-021-01036-7

**Published:** 2021-04-12

**Authors:** Emma Guerin, Andrey N. Shkoporov, Stephen R. Stockdale, Joan Colom Comas, Ekaterina V. Khokhlova, Adam G. Clooney, Karen M. Daly, Lorraine A. Draper, Niamh Stephens, Dimitri Scholz, R. Paul Ross, Colin Hill

**Affiliations:** 1 APC Microbiome Ireland, University College Cork, Cork, Ireland; 2 School of Microbiology, University College Cork, Cork, Ireland; 3 Conway Institute of Biomolecular and Biomedical Research, University College Dublin, Belfield, Dublin 4, Ireland; 4 Teagasc Food Research Centre, Moorepark, Fermoy, Co. Cork Ireland

**Keywords:** Bacteriophages, crAssphage, crAss-like phages, Human gut phageome, Human microbiome, Phage-host interactions

## Abstract

**Background:**

The gut phageome comprises a complex phage community of thousands of individual strains, with a few highly abundant bacteriophages. CrAss-like phages, which infect bacteria of the order Bacteroidales, are the most abundant bacteriophage family in the human gut and make an important contribution to an individual’s core virome. Based on metagenomic data, crAss-like phages form a family, with four sub-families and ten candidate genera. To date, only three representatives isolated in pure culture have been reported: ΦcrAss001 and two closely related phages DAC15 and DAC17; all are members of the less abundant candidate genus VI. The persistence at high levels of both crAss-like phage and their Bacteroidales hosts in the human gut has not been explained mechanistically, and this phage-host relationship can only be properly studied with isolated phage-host pairs from as many genera as possible.

**Results:**

Faeces from a healthy donor with high levels of crAss-like phage was used to initiate a faecal fermentation in a chemostat, with selected antibiotics chosen to inhibit rapidly growing bacteria and selectively enrich for Gram-negative Bacteroidales. This had the objective of promoting the simultaneous expansion of crAss-like phages on their native hosts. The levels of seven different crAss-like phages expanded during the fermentation, indicating that their hosts were also present in the fermenter. The enriched supernatant was then tested against individual Bacteroidales strains isolated from the same faecal sample. This resulted in the isolation of a previously uncharacterised crAss-like phage of candidate genus IV of the proposed Alphacrassvirinae sub-family, ΦcrAss002, that infects the gut commensal *Bacteroides xylanisolvens*. ΦcrAss002 does not form plaques or spots on lawns of sensitive cells, nor does it lyse liquid cultures, even at high titres. In keeping with the co-abundance of phage and host in the human gut, ΦcrAss002 and *Bacteroides xylanisolvens* can also co-exist at high levels when co-cultured in laboratory media.

**Conclusions:**

We report the isolation and characterisation of ΦcrAss002, the first representative of the proposed Alphacrassvirinae sub-family of crAss-like phages. ΦcrAss002 cannot form plaques or spots on bacterial lawns but can co-exist with its host, *Bacteroides xylanisolvens*, at very high levels in liquid culture without impacting on bacterial numbers.

**Video abstract**

**Supplementary Information:**

The online version contains supplementary material available at 10.1186/s40168-021-01036-7.

## Background

It is over a century since the discovery of bacteriophages (phages), viruses capable of infecting bacterial cells. Phages are almost certainly the most abundant biological entities on Earth with an estimated total count of 10^31^ virions in the biosphere, potentially outnumbering their bacterial hosts by a factor of 10 [1, 2]. In recent years, interest in phage communities residing in the human gut (the gut phageome) has greatly increased due to the growing awareness of the role of gut microbiome in human health and a resurgence in interest in phage therapy [3, 4]. Recent reports also suggest that the human gut phageome may play a role in human health and disease [5–13].

The viral (which is overwhelmingly phage) fraction of the human gut microbiome remains its most elusive component [14, 15]. In human faeces, the viral load has been estimated at only 10^9^–10^10^ virus-like particles (VLPs) g^-1^, meaning that unlike in other environments where phage dominate, phage in the human gut are outnumbered by their bacterial hosts [3]. Advances in sequencing technology have allowed us to generate vast numbers of viral sequences, but the majority are poorly annotated and unclassified taxonomically due to the lack of homology with known viruses in current databases. These sequences have been termed viral “dark matter” and can constitute up to 90% of total virome reads [16]. This is one of the most significant bottlenecks in phage research. Perhaps the best example are the crAss-like phages, the most abundant phages in the human gut, but no representative had ever been cultured and it was only identified by a database-independent approach in 2014 [17].

The prototypical crAssphage (p-crAssphage) is a 97 kb dsDNA phage of the Caudovirales order that was detected through cross-assembly (using crAss software) of human gut viral reads from metagenomic data. Intriguingly, this phage shared no homology with any other virus in databases at the time of its discovery, despite its extraordinary abundance (up to 90% of all phages in some individuals) [17]. Protein sequence-based analysis of the p-crAssphage demonstrated that this phage is the founder member of a family-level group of ‘crAss-like phages’ and was predicted to have a podovirus-like morphology [18]. We have proposed a taxonomic system for crAss-like phages from the human gut with four candidate subfamilies (Alpha-, Beta-, Gamma-, and Deltacrassvirinae) and ten candidate genera (I–X), with p-crAssphage belonging to genus I of the proposed Alphacrassvirinae group. We then confirmed the predicted podovirus-like morphology by transmission electron microscopy of a crAss-like phage rich faecal filtrate and followed this with the isolation in culture of the first representative member of the family, ΦcrAss001 (candidate genus VI, subfamily Betacrassvirinae) [19, 20].

Although crAss-like phages are largely gut associated, they have also been detected in other diverse samples such as animal litter, surface/ground water, and termite gut [18, 21]. In humans, the relative abundance of this phage family in the gut can be as high as 90% of the total viral load in some individuals. The geographical spread of this phage family has also been confirmed with p-crAssphage for example, being largely absent from hunter-gatherer gut populations compared to industrialized populations [19, 22–24]. This may be due to differences in dietary habits and bacteriome compositions. Our recent results showed that 77% of healthy Western adults carry one or more representatives of this phage family, although at widely variable abundances [19]. Despite the fact that hundreds of crAss-like phage genomes have been identified in silico, their bacterial hosts remain to be determined [17, 25]. The host phylum was hypothesised to be Bacteroidetes through co-abundance analysis, the presence of BACON-domain-containing proteins which are very characteristic of this phylum and partial matches in CRISPR-spacer sequences [17]. ΦcrAss001, that infects *Bacteroides intestinalis*, became the first crAss-like phage to be isolated in pure culture in 2018 [20].

Here, we present the isolation and characterisation of ΦcrAss002 that infects the gut commensal *Bacteroides xylanisolvens*. This is the first member to be isolated from the proposed sub-family Alphacrassvirinae. Initial attempts to isolate crAss-like phages using traditional methods, such as screening of crAss-rich faecal samples using plaque or spot assays, proved unsuccessful. ΦcrAss002 was isolated following ex vivo enrichment in a faecal fermentation using antibiotics to selectively promote the growth of Bacteroidales, followed by liquid culturing, metagenomic sequencing, in silico analyses and quantitative real-time PCR. Biological characterisation of ΦcrAss002 confirmed that this phage shares multiple traits with ΦcrAss001, while also possessing several unique characteristics.

## Results

### Faecal fermenter enrichment workflow

A faecal fermentation was initiated using faeces from an individual (donor 924) previously identified as a persistent carrier of multiple crAss-like phages. The fermentation was performed under anaerobic conditions with vancomycin and kanamycin added in an attempt to suppress the growth of Gram-positive and facultative anaerobic bacteria and favour the strictly anaerobic Gram-negative Bacteroidales. (Additional file 1: Figure S1) depicts the workflow.

**Fig. 1:**
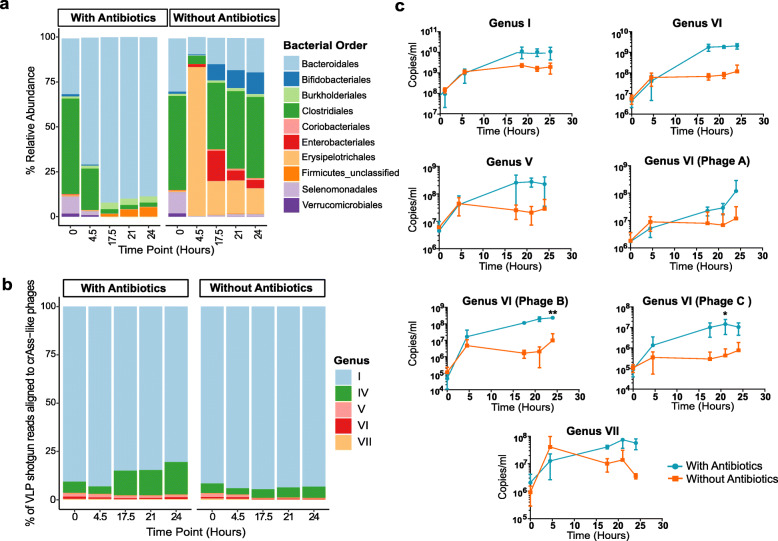
The effect of antibiotic selective enrichment in faecal fermenter on the abundance of bacterial orders and the parallel effect on different crAss-like phage abundances. **a** The mean (across three experimental runs) relative abundances of the key bacterial orders under both conditions tested (only orders with relative abundance of 1% in any of the samples are shown). **b** The mean relative abundance of crAss-like phage contigs per genus as a percentage of total crAss-like reads. The crAss-like phage contigs are coloured based on candidate genus. CrAss-like phages that resolve into five of the ten crAss-like family candidate genera were detected. **c** Absolute quantification of each crAss-like phage detected using qPCR with phage or genus-specific primers targeting a segment of the terminase or primase gene. The error bars indicate standard deviation (*n* = 3). The asterisks denote statistical significance in *p* value (*p value ≤ 0.01; ** *p* value ≤ 0.001)

16S rRNA gene analysis of the bacterial composition of the faecal fermentates confirmed a significant increase in the relative abundance of Bacteroidales in the presence of antibiotics (Fig. 1a). After 4.5 h, the fermenter vessel was dominated by members of this order and remained so for the remainder of the run. *Bacteroides* and *Parabacteroides* dominated at the genus level with the former representing as much as 75% of 16S rRNA gene reads. The vessels without added antibiotics were largely dominated by Gram-positive bacterial orders. There was a rapid expansion of Erysipelotrichales after 4.5 h, but by 17.5 h, the relative abundance of this order decreased and Clostridales once again became the dominant order. Diversity indices further highlight the shift in the overall bacterial communities under selective conditions (Additional file 2: Figure S2a).

### Enrichment of crAss-like phages

Shotgun metagenomic sequencing data, generated from viral enriched DNA extracted from three separate fermentation runs for each condition, was examined for the presence of crAss-like phages. Assembled viral contigs were compared against an in-house database of 249 crAss-like phages using BLASTn [19]. This database includes seven crAss-like phages previously detected in donor 924 6 months prior to donating a sample for this study [19]. Six of the original seven crAss-like phages could be detected but one phage (originally denoted as Fferm_ms_1 of candidate genus II) was no longer detectable. However, a previously undetected crAss-like phage of candidate genus VII was now present, meaning that five different crAss-like phage genera were represented in the sample. The relative abundance of all the crAss-like phages represented 38% of the total viral reads derived from VLPs. The relative abundance of each of the five genera was calculated as a percentage of the crAss-like phage reads (Fig. 1b). In the presence and absence of antibiotics, the vessels were dominated by a phage of candidate genus I (p-crAssphage). The relative abundance of a phage of candidate genus IV was found to increase under the selective conditions. In line with similar alterations in the bacteriome, we observed a reduction in virome alpha-diversity, species richness, and evenness when antibiotics were included in the fermenter (Additional file 2: Figure S2b).

qPCR directed at the conserved terminase or primase genes was performed to determine an approximate titre in copies/ml of the seven crAss-like phages (Fig. 1c). Primers were either genus specific (genera I, IV, and V) or phage strain-specific (genera IV and VII) depending on the level of homo- or heterogeneity of the terminase gene sequence in each genus (or primase in the case of candidate genus VII). For each candidate genus, higher phage titres were detected in the presence of antibiotics, although to varying extents. Candidate genus I was 6-fold higher in titre in comparison to the control vessel by the end of the fermentation, candidate genus IV 17-fold, candidate genus V 8-fold, candidate genus VI phage A 10-fold, candidate genus VI phage B 24-fold, candidate genus VI phage C 14-fold and candidate genus VII 5-fold higher. The statistical significance of the differences between the highest titres attained for each phage under both conditions tested was examined by a two-tailed paired *t* test. This showed that a statistically significant titre difference occurred for three of the phages: candidate genus IV and candidate genus VI phage B (*P* value ≤ 0.001) and candidate genus VI phage C (*P* value ≤ 0.01).

### Screening a faecal sample for potential phage hosts

The same crAss-rich faecal sample was plated on antibiotic agar selective for Bacteroidetes, and 48 colonies were chosen based on variations in colony morphology. Sequencing of the 16S rRNA gene fragment assigned the 48 isolates to six species: *Bacteroides uniformis*, *Bacteroides ovatus*, *Bacteroides dorei*, *Bacteroides fragilis*, *B. xylanisolvens* and *Parabacteroides distasonis* (Additional file 3: Table S1). The crAss-like phage-enriched fermentate was added to a pure culture of each of the 48 strains, and following incubation anaerobically at 37°C genus- or phage-specific qPCR was used to detect increases in individual phage strains (Fig. 1c). We detected propagation of a crAss-like phage of candidate genus IV on *B. xylanisolvens* APCS1/XY following three consecutive sub-cultures. Despite the efficient propagation of the phage during this and subsequent enrichment, the liquid culture failed to clear. When faecal filtrate prepared from subject ID:924 faeces prior to fermentation was spotted onto a lawn of *B. xylanisolvens* APCS1/XY no zone of clearing or individual plaques were observed.

### Genome analysis of ΦcrAss002

**Fig. 2:**
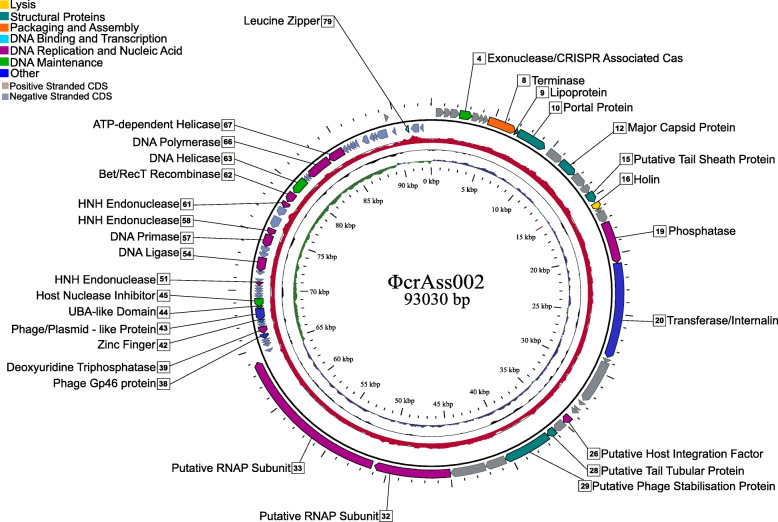
Circular genome map of the ΦcrAss002 genome. The innermost ring (blue; positive strand, green; negative strand) depicts G + C skew, the central ring (black) shows G + C content and the outer ring (red) highlights Illumina read coverage along the genome. The outermost circle shows coding genes (CDS) which are labelled on HHpred function predictions. CDS are coloured based on general function which corresponds to the legend. No function predictions were possible for genes which are unlabelled

Shotgun sequencing of the phage propagated in pure culture confirmed the isolation of a novel crAss-like phage, designated ΦcrAss002, with a circular genome of 93,030 bp (NCBI GenBank MN917146) (Fig. 2). ΦcrAss002 genome has 81 protein-coding genes in two oppositely orientated gene modules (possibly two transcriptional units) but less than half could be assigned a function (Additional file 4: Table S2). No genes for lysogeny functions were identified, and we did not detect integrated copies of this or related phages in bacterial genomes (NCBI RefSeq). Module 1 spanning 45–93kb is largely dominated by functions associated with replication and nucleic acid metabolism, whereas module 2 spanning 0–45kb includes phage structural genes, as well as those encoding lysis and packaging functions. Two large genes (gp32 and gp33) located at the beginning of module 1 are predicted to encode RNA polymerase subunits. The ΦcrAss001 genome also has large genes in a similar location that were assigned the same function [20]. The G+C content of the ΦcrAss002 genome is 31.92 mol%, approximately 10 mol% lower than the host G+C content of 42.24 mol%. The tail components and the receptor binding proteins remain to be identified, but are likely to be associated with gp11, 13–15, and/or 17–31 [26, 27]. The DNA packaging mechanism of ΦcrAss002 was predicted to be headful packaging with terminase initiation occurring at a *pac* site, and so the packaged genomes are circularly permuted with redundant termini.

**Fig. 3:**
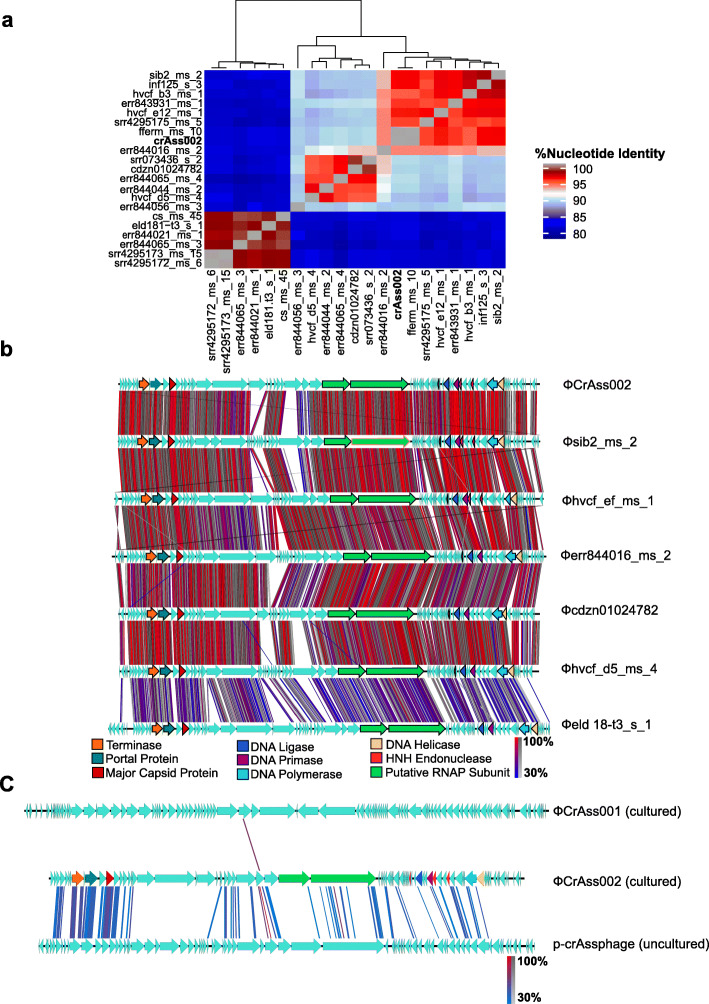
In silico characterisation of ΦcrAss002. **a** To examine relatedness of ΦcrAss002 with twenty other phages of candidate genus IV identified in silico, a heatmap was generated based on average nucleotide identity (ANI). **b** Whole genome comparisons of ΦcrAss002 and a subset of related phages to highlight synteny and genome organisation. In decreasing order from the top are phages with higher to lower ANI/relatedness. Genes with predicted functions are colour-coded based on generalised function. Areas of tBLASTx homology between the genomes are highlighted. **c** Whole genome comparison of ΦcrAss002 with ΦcrAss001 (sequence from pure isolate) and the prototypical crAssphage (sequence solely in silico) to examine synteny and homology. Regions of homology (tBLASTx) are highlighted

The average nucleotide identity (ANI) was determined between ΦcrAss002 and twenty other previously identified genomes of candidate genus IV [19] for which additional metadata is available (Fig. 3a, Additional file 5: Table S3). There are three obvious clades with >95% nucleotide similarity, recommended by The International Committee on Taxonomy of Viruses (ICTV) as the cut-off level for species [28]. Therefore, amongst the crAss-like candidate genus IV phage sequences, there are potentially four species, with err844056 potentially forming its own species. Genome comparison of a subset of these phages, with different degrees of relatedness, revealed a high degree of genome synteny (Fig. 3b). Phages are in descending order of decreasing ANI in relation to ΦcrAss002. Even the most distant representative shown here, φeld18-t3_s_1, shares a genome organisation highly syntenic to that of ΦcrAss002.

The genomic synteny of ΦcrAss002, ΦcrAss001 and p-crAssphage (prototypical crAssphage generated only from in silico data were also compared (Fig. 3c). ΦcrAss002 shares no significant tBLASTx homology with ΦcrAss001, but there is much greater homology and synteny between ΦcrAss002 and p-crAssphage (candidate genus I), which could be expected as these phages both belong the proposed Alphacrassvirinae sub-family.

**Fig. 4:**
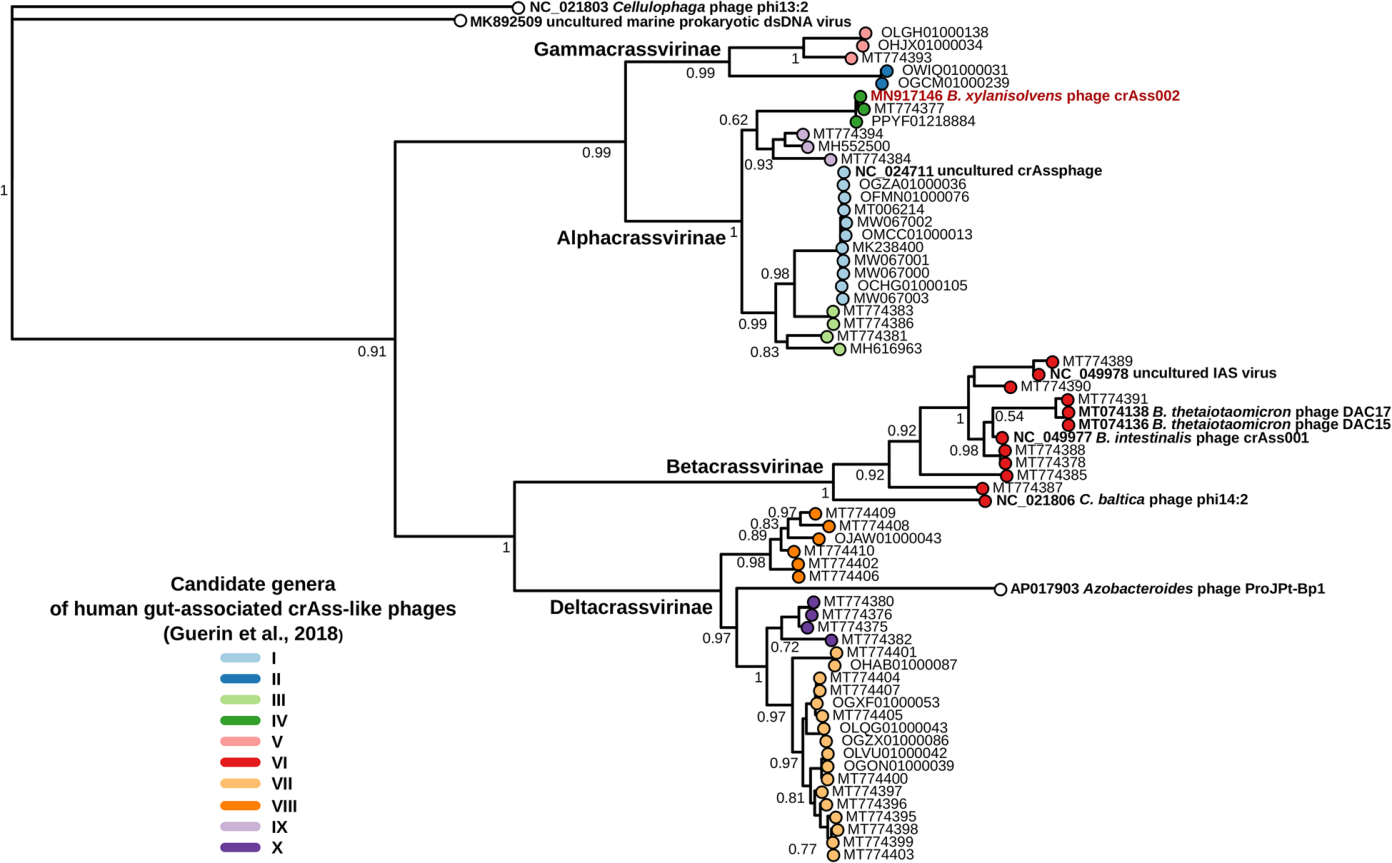
Phylogenetic tree of major capsid proteins encoded by published complete genomes of crAss-like phages. Protein sequences were aligned using MUSCLE, approximately-maximum-likelihood phylogenetic trees were generated using FastTree. Branch support values calculated using SH-test. Tree tip colours correspond to candidate genera as proposed in [19]. ΦcrAss002 label is highlighted in red. Previously well-characterised uncultured phage genomes and cultured isolates are highlighted in boldface font. Accession numbers of genomes in NCBI GenBank/RefSeq/WGS databases are provided

To investigate relationships between ΦcrAss002 and more distantly related members of crAss-like phage family, we performed multiple alignments of major capsid proteins (gp12 in ΦcrAss002) and large terminase subunits (gp8) encoded by representative complete genomes from various clades within the family. The resulting phylogenetic trees clearly place ΦcrAss002 within candidate genus II of the proposed sub-family Alphacrassvirinae (Fig. 4, Additional file 6: Figure S3).

### Biological characterisation of ΦcrAss002

**Fig. 5:**
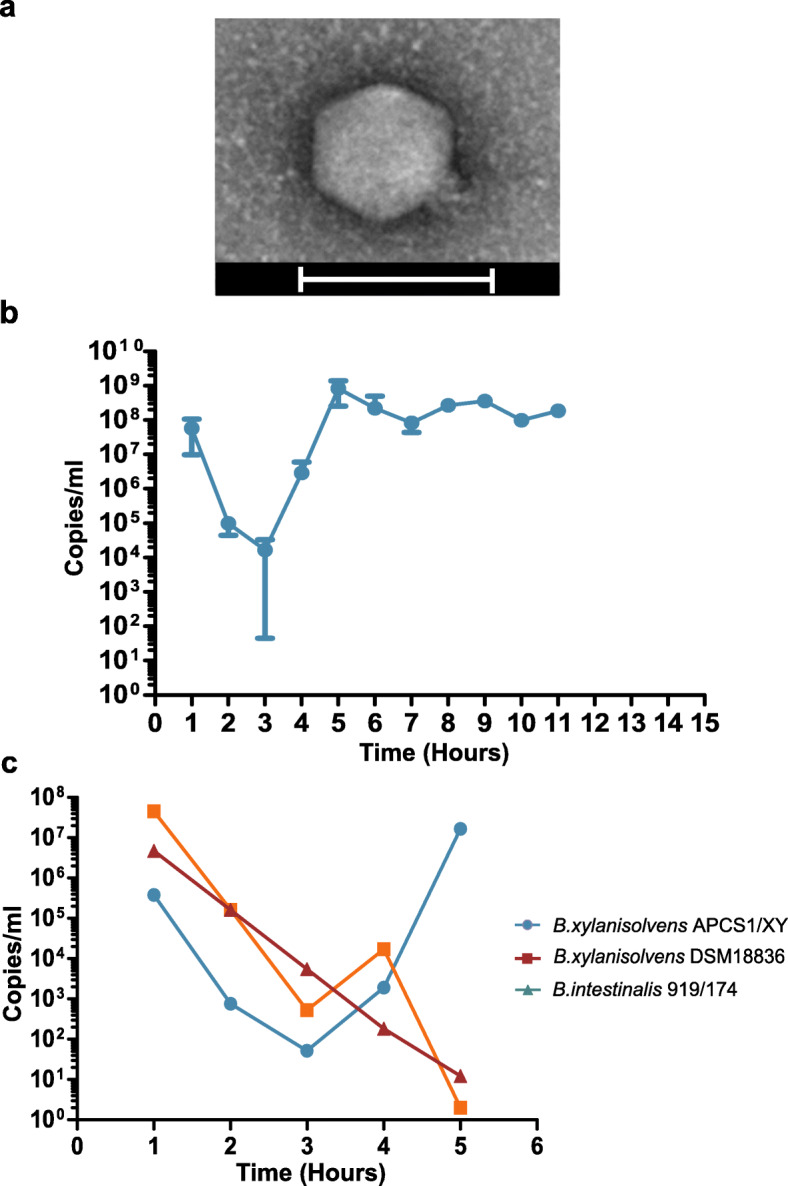
Biological characterisation of ΦcrAss002. **a** Transmission electron micrograph, generated from ΦcrAss002 enriched lysate, stained with uranyl acetate. Micrographs show podovirus-like virions with a diameter of ~77 nm and a simple tail structure. Scale bars represent 100 nm. **b** In vitro propagation of ΦcrAss002 over 11 days. Titre quantification was performed using qPCR. **c** Investigation of the ability of ΦcrAss002 to propagate on commercial *B. xylanisolvens* DSM18836 and the only other confirmed crAss-like phage host to date, *B. intestinalis* 919/174 via liquid propagation over 5 days. Error bars indicate standard deviation (*n* = 3)

Transmission electron microscopy confirmed that ΦcrAss002 has a Podovirus morphology with a capsid diameter of 77.0 ± 2.0 nm and tail of 18.1 ± 2.3 nm (Fig. 5a). Unlike ΦcrAss001, the tail structure is simple and has no obvious appendages. Plaque assays and spot assays were performed using enriched cultures of ΦcrAss002. Plaques failed to form despite testing several media modifications, and spots with concentrated phage suspensions were opaque and only barely visible. The clarity of spots was variable between independently generated overnight cultures of *B. xylanisolvens* APCS1/XY. Furthermore, the phage only propagated if co-cultured for a minimum of 3–5 days.

To examine the growth dynamics of ΦcrAss002, the phage and host were serially propagated via daily sub-culturing over 11 days. The phage load was quantified daily in copies/ml using qPCR. The phage failed to propagate between day 1 and day 3 during which a reduction in titre of about 4 to 6 logs occurred. This is largely consistent with the dilution effect of a 1% inoculum. After day 3, the titre increased until it stabilised at approximately 10^8^ copies/ml from day 5 onwards (Fig. 5b). The observed stability is consistent with the persistence of crAss-like phages observed in human gut viromes over time [4]. The commercially available strain *B. xylanisolvens* DSM18836 failed to support replication of ΦcrAss002, as did the ΦcrAss001 host, *B. intestinalis* 919/174 (Fig. 5c). When *B. xylanisolvens* APCS1/XY was plated on an agar plate coated with a high-density preparation of ΦcrAss002, 76% of bacterial cells gave rise to a colony. This suggests that a dominant fraction of the population does not support phage replication (perhaps due to phase variation). Ten of these colonies were triple streaked and tested for the presence of episomally replicating or integrated copies of ΦcrAss002. PCR confirmed all clones were phage negative. When isolates were tested with the phage by spot assay, the spots presented with varying turbidity.

### Characterisation of the ΦcrAss002 host *Bacteroides xylanisolvens* APCS1/XY

The genome of *B. xylanisolvens* APCS1/XY consists of a single circular chromosome (6,461,058 bp, GenBank CP042282) and two circular plasmids (pBXS1-1, 5,595 bp, GenBank CP042281 and pBXS1-2, 4,148 bp, GenBank CP042283). A considerable number of elements were identified in the genome that could potentially drive phase variation of surface structures and contribute to phage resistance/sensitivity. These included multiple genes coding for site-specific tyrosine recombinases (9), tyrosine-type DNA invertases (4) and site-specific integrases (28) (Additional file 7: Figure S4a). In certain cases, these genes were in proximity to genes encoding bacterial surface molecules that have been previously identified as being subjected to phase variation and affecting surface composition and phage sensitivity in *Bacteroides* [29]. These bacterial features included nutrient uptake genes such as the products of the *sus* gene family, genes coding for TonB-dependent nutrient transporters or capsule polysaccharide biosynthesis genes [29–32]. Examples of cell-surface associated genes co-localised with site-specific recombinases were observed at the following loci: FNQN58_01735-01770 (TonB gene family + tyrosine-type recombinase/integrase), FNQN58_07900-07925 (TonB/Sus/Rag gene family + tyrosine-type recombinase/integrase) and FNQN58_12195-12225 (lipopolysaccharide and capsule biosynthesis + tyrosine-type recombinase).

In order to obtain preliminary evidence of phase variation in *B. xylanisolvens* APCS1/XY associated with dynamic recombinations in the genome, we performed analysis of individual Oxford Nanopore reads. Reads were aligned to the assembled chromosome scaffold and recombination hotspots, indicating potential phase-variable loci were identified (Additional file 7: Figure S4b-d). Interestingly, many of the recombination hotspots overlapped with or occur in proximity to genes that function as Sus or TonB family transporters or receptors, site-specific integrases, restriction endonucleases or transposases. For example, a recombination hotspot that extends from genome position 6151000–6152000 bp overlaps a TonB-dependent receptor and occurs in proximity to RagB/SusD family nutrient uptake outer membrane protein encoding genes and site-specific integrase. Another example extends from 909000–910000 bp overlapping a site-specific integrase which occurs upstream of a capsule biosynthesis gene. The hotspot identified across genome position 916000–917000 bp overlaps a glycosyltranserase gene which is in proximity to lipopolysaccharide synthesis genes.

Other features of interest encoded by the *B. xylanisolvens* APCS1/XY genome were over 139 transposases associated with thirteen or more insertion sequence (IS) families, the presence of three xylanase characteristic of this bacterial species, over one hundred *sus*/*tonB-*associated genes and three capsule polysaccharide biosynthesis operons (Additional file 7: Figure S4a). Annotation of plasmids pBXS1-1 and pBXS1-2 showed that both carry genes coding for toxin-antitoxin systems which may have a role in phage defences by mechanisms such as abortive infection. Other roles of these systems include post-segregational killing or persistent formation which allows transient physiological changes that increase tolerance to antibacterial substances such as antibiotics [33]. Plasmid pBXS1-2 also carries a gene that encodes for vesicle formation. This may be linked to the outer membrane vesicle-like structures (OMVs) observed in micrographs from cross-sections of soft agar lawns prepared with cultures of *B. xylanisolvens* APCS1/XY, with and without ΦcrAss002 exposure (Additional file 8: Figure S5). The formation of such vesicles from the outer membrane occurs naturally among Gram-negative bacteria and they are thought to have multiple roles including secretion and transport of soluble and insoluble molecules, DNA transfer, stress adaptation, virulence and phage defence [34, 35]. The precise role of the vesicles observed on the surface of *B. xylanisolvens* APCS1/XY cells remains to be elucidated.

### Co-cultivation of ΦcrAss002 and *Bacteroides xylanisolvens* APCS1/XY

**Fig. 6:**
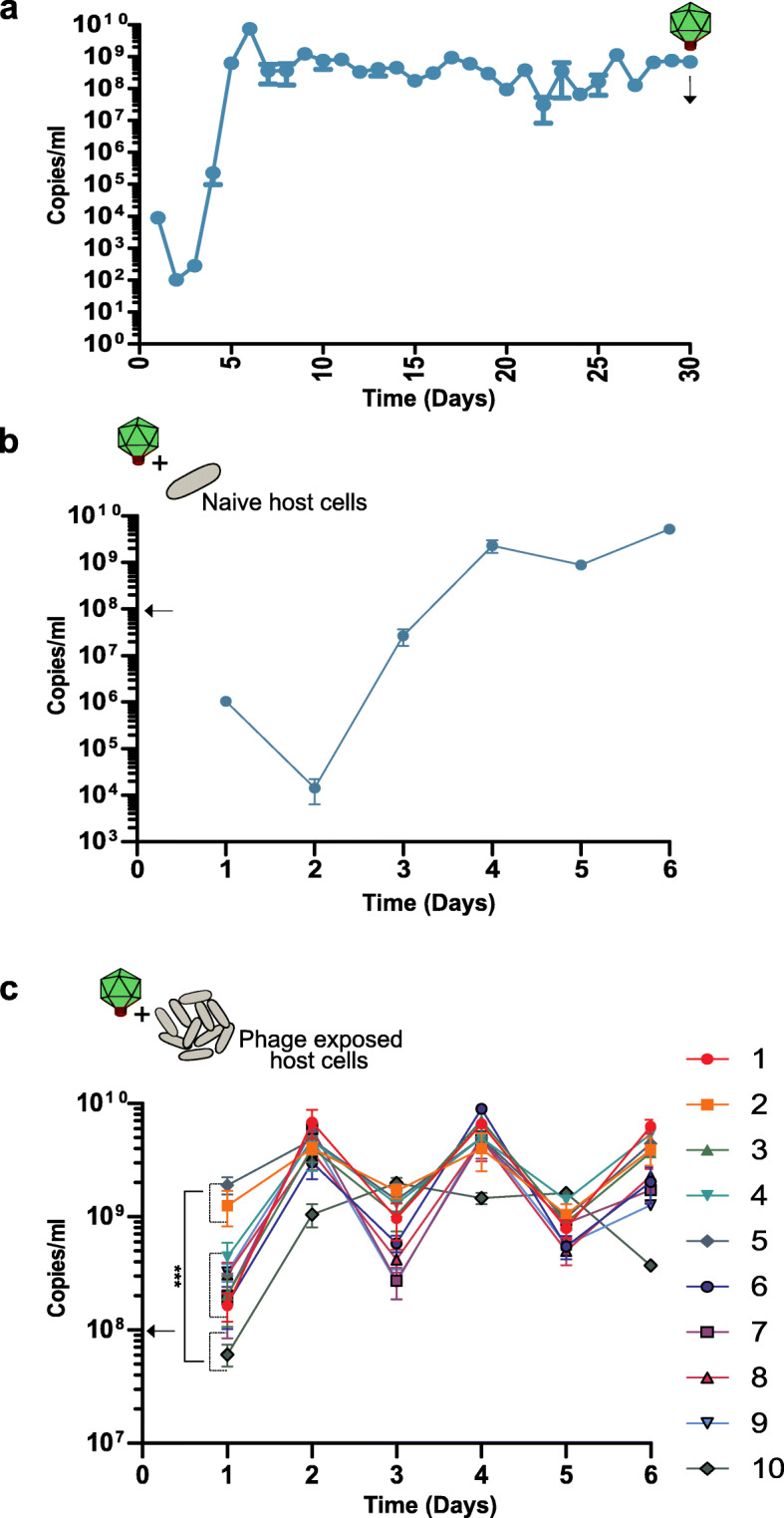
Continuous co-culture of ΦcrAss002 and *Bacteroides xylanisolvens* APCS1/XY. **a** Serial co-culturing of ΦcrAss002 over 30 days*.* **b** Serial propagation of the phage on naïve host cells (absent of phage exposure for ~10 generations). **c** Equivalent experiment using ten *B. xylanisolvens* cultures with recent phage exposure. ΦcrAss002 titre is shown in copies/ml, determined by absolute qPCR. Statistical analysis was performed by the one-way ANOVA (*p* < 0.001) with Tukeys as post-test comparing titres. A statistical significance of the difference between the lowest titre and the highest titre sustained shown (*p* < 0.001***). The arrows indicate the approximate titre ΦcrAss002 at the initiation of each propagation cycle. The error bars represent standard deviation (*n* = 3)

We have already described the phenomenon in which ΦcrAss002 fails to propagate for several days in the presence of its host, before accumulating to and maintaining high levels (Fig. 5b). This suggests that either the phage or the host has undergone some adaptation within the first days of propagation. This was confirmed via serial co-culturing over 30 days in which the same phenomenon was observed. Following recovery and stabilisation, the phage propagated at approximately 10^8^ copies/ml for 21 days (Fig. 6a). Phage lysate collected at the end of the 30 days was then propagated on a naïve host (a bacterial culture which has not been in recent contact with the phage) and once again there was an initial drop in titre, consistent with a dilution effect, before recovery and maintenance at high titres (Fig. 6b). This suggests that phage variants have not been selected and that bacterial host adaptation may be responsible for the stable co-propagation. To examine this, ten individual colonies were selected from the bacterial pellet formed after centrifugation of the 6-day co-culture on naïve cells (grown in the presence of phage) and used as the starting material for another phage co-culture cycle. Three types of behaviour were observed. Two clones immediately supported ΦcrAss002 propagation at high titres, whereas the phage titre dropped significantly in the presence of one clone (Fig. 6c). The remaining seven clones gave an intermediate response. By day 2, all cultures supported a high titre of ΦcrAss002 of ~10^9^ copies/ml. On naïve cells, 4–5 days of co-culturing is required to achieve similar titres. This suggests that the bacterial population is heterogenous in terms of its phage permissiveness and that counterintuitively the presence of the phage selects for phage sensitive host variants. When ΦcrAss002 was spotted on lawns of the phage exposed clones, spot turbidity was reduced in varying amounts compared to spots on lawns of naïve cells.

### Impact of crAssphage on hosts in a defined community

**Fig. 7:**
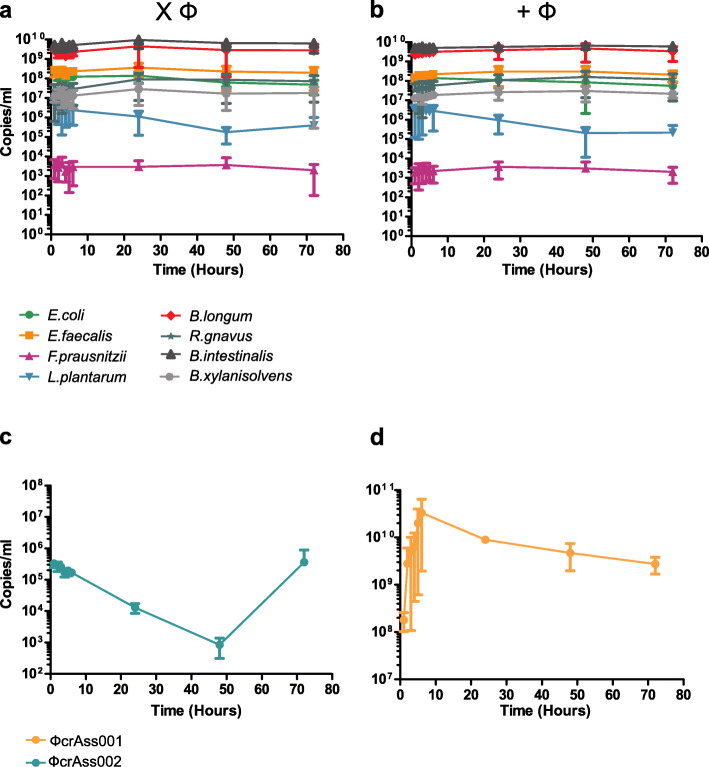
The impact of ΦcrAss001 and ΦcrAss002 on host counts in a defined bacterial community. Continuous fermentations were performed in parallel with and without phage addition. Respective hosts were included *B. xylanisolvens* (ΦcrAss002) and *B. intestinalis* (ΦcrAss001). Absolute quantification, by qPCR, was performed on total DNA. **a** Absolute quantification of the bacterial community structure without phage addition. **b** Equivalent graph showing the community structure in the presence of the phages. **c** The titre and propagation dynamic of ΦcrAss002. **d** Equivalent graph for ΦcrAss001. The error bars represent standard deviation (*n* = 3)

A continuous fermentation model was initiated in triplicate with a defined bacterial community constructed from eight different species representing a simplified human microbiota consortium (SIHUMI) (Fig. 7). Following inoculation at similar levels, a community structure formed within hours that remained stable for 72 h (Fig. 7a). When ΦcrAss002 and ΦcrAss001 were added from time point 0, there was no impact on either the levels of their individual hosts, or on the community structure (Fig. 7b). ΦcrAss002 levels decreased as before consistent with a washout due to media replacement, before recovering within 72 h (Fig. 7c). ΦcrAss001 achieved high levels within a few hours and were maintained at high titres thereafter (Fig. 7d).

## Discussion

Further advances in understanding the biology of the human gut phageome will depend on the isolation, propagation, and characterisation of individual phage-host pairs. This is particularly true for the most abundant representatives of this viral community, the crAss-like phages. With the exception of ΦcrAss001 and two close relatives of this phage, DAC15 and DAC17, all other crAss-like genomes described to date are the result of composite assemblies and have never been propagated in pure culture in a laboratory [4, 6, 17–20, 23, 36–40]. Biological characterisation of ΦcrAss001 revealed several intriguing traits. Although shown to be virulent and capable of forming plaques on agar plates, it fails to clear liquid cultures of its host where both phage and host can stably co-exist and propagate to high levels [20]. CrAss-like phages also form part of the personal persistent virome (PPV), a consistently present, individual-specific core of mostly virulent phages in the viromes of healthy individuals [4, 13]. In addition, crAss-like phages have been demonstrated to engraft and persist in the microbiome of faecal microbiota transplantation recipients and can undergo vertical transmission between mother and infant [36, 38, 40]. This suggests that ecological models yet to be characterised are at play in the human gut that allow this persistence. It may be that virulent phages employ a “piggyback-the-winner” strategy to take advantage of the success of well-adapted bacterial hosts in the gut. Originally, this model was described by Rohwer and colleagues proposing that in thriving bacterial populations with a low virus-to-microbe ratio (VMR), phages can make a lytic to lysogenic lifestyle switch to “piggyback on” the success of their host and thus ensure their maintenance in the ecosystem [41, 42]. In the context of the human gut, similar strategies may be employed that do not involve lysogeny but allow virulent phages persist in the human gut. The human gut is thought to have a VMR of ~0.1:1, which is lower than the VMR predicted in other ecosystems [3]. Virulent gut phages may have evolved less aggressive infection strategies to ensure efficient and sustained replication without host elimination thus ensuring their persistence in the gut. Elucidation of the mechanisms behind this persistence requires further investigation.

Isolation of these dominant gut bacteriophages is a challenge given that they are likely to be specialists in terms of their host range, and the high levels of variation observed in their predicted receptor-binding proteins [20]. Furthermore, many anaerobic gut bacteria are difficult to cultivate and variations in host sensitivity/resistance may make it difficult to identify phage host pairs by standard agar-based methods such as plaquing and spot assays. The isolation of further crAss-like phages will be important in helping us understand their interaction with their host(s) and how certain phages can persist in the human gut over such extensive time periods.

The overall objective of this study was the isolation of novel phages from the gut of an individual previously identified as being rich in several different crAss-like phages. The antibiotic-driven crAss-like phage enrichment implemented in this study confirmed that suitable hosts for crAss-like phage propagation were present and viable in the faecal samples. This led to the isolation of ΦcrAss002, a novel member of the crAss-like phage family isolated in pure culture. ΦcrAss002 infects *B. xylanisolvens*, a Gram-negative, strictly anaerobic, non-pathogenic, xylan-degrading bacterium, which was first isolated from human faeces in 2008 [41, 42]. Recently, *B. xylanisolvens* has been demonstrated to be capable of boosting production of natural TFα sugar antigen-specific IgM antibodies in healthy humans, an antibody response believed to be involved in cancer immune surveillance [43]. Due to the associated beneficial properties of this bacterial species, *B. xylanisolvens* DSM23964 is one of the few bacterial strains approved by the European Food Safety Authority for applications in a novel food; heat-treated milk products fermented with the strain in a non-viable form, under the Novel Food Regulation No 258/97 [44].

ΦcrAss002 is a member of candidate genus IV of the Alphacrassvirinae sub-family, according to our recently proposed taxonomic scheme for gut-associated crAss-like phages [19]. The first crAss-like phage isolated, ΦcrAss001, infects *B. intestinalis* and is a member of the more heterogenous candidate genus VI of the sub-family Betacrassvirinae [20]. The isolation of two crAss-like phages from the same genus VI has been reported, DAC15 and DAC17, that both infect *Bacteroides thetaiotaomicron* [45]. Detailed biological characterisation of these two phages, isolated from sewer-adjacent pond water collected in Bangladesh will provide further insights into this phage family. ΦcrAss002 is more closely related to p-crAssphage, the founder member of the family and most abundant bacteriophage in the Western populations, which also belongs to the Alphacrassvirinae sub-family (Fig. 3c). The cultured crAss-like phages share a podovirus-like morphology, but the tail structure of ΦcrAss001 appears to be more elaborate than that of ΦcrAss002 (Fig. 5a) [20]. ΦcrAss001 produces large plaques whereas ΦcrAss002 does not and even at high titre only forms opaque zones of clearing when spotted in high concentration on a lawn of its host bacterium. Both phages also employ different mechanisms for DNA packaging (short direct terminal repeats in ΦcrAss001 versus pac-type headful packaging in ΦcrAss002). Despite their apparent differences, both phages share notable similarities (Additional file 9: Table S4). Both phages infect *Bacteroides* hosts, appear to be specialists in host range, and neither possesses lysogeny-associated genes nor are they able to form stable lysogens or pseudolysogens. Their genomes also differ in G + C content by about 10% mol in comparison to their host genomes. While ΦcrAss001 and ΦcrAss002 are virulent in nature, both fail to clear liquid cultures yet still reach high titres. Intriguingly, both can co-exist at high levels with their host over prolonged periods.

It has become apparent that many virulent phages persist at high levels in concert with their bacterial hosts. This has been indicated by observations in multiple studies [20, 46–49]. Overall, the “kill-the-winner” dynamics have not been observed in the human gut virome, at least at the level of resolution of genus or species taxonomic levels [4, 14, 50–52]. The observed persistence of the crAss-like phages is consistent with an in-depth longitudinal study of the virome which followed ten healthy individuals over a 1-year period. The stable abundant component of a healthy virome largely consists of virulent phages (including crAss-like phages) with a minority of temperate phages [4]. This study strongly supports the notion that phage communities employ strategies that allow stable co-existence with their hosts.

Continuous co-culture of ΦcrAss002 revealed that propagation does not occur efficiently on initial host contact. Following a period in which no replication is observed, phage titre recovers and stabilizes (Fig. 6a, b). This suggests that either growth in the presence of phage, or, potentially, other independent variables (e.g. nutrient availability affecting expression of surface proteins) gradually select for a phage permissive population composition in these particular experimental settings. When added to a simplified bacteria community grown in a chemostat host levels are unaffected by the presence and propagation of the phages (Fig. 7b). Similar findings were observed for phages infecting enteroaggregative O104:H4 *Escherichia coli* in a conventional mouse model. These phages, isolated from sewage, were able to propagate stably and continuously over a number of weeks in vivo. Interestingly, faecal bacterial counts for phage treated and non-phage treated mice remained the same despite phage propagation [46]. In the fermenter, no knock-on effects were observed for off-target community members. This is both supported and contradicted by other in vitro and in vivo studies [49, 53, 54]. The observed stability and persistence may be due “piggyback-the-winner” style dynamics which describes the way in which phages can adopt less disruptive infection strategies and “piggyback” on the success of their bacterial host in an ecosystem [55, 56]. This allows more efficient replication and permits co-existence with that cognate host that would not be achieved with aggressive lytic replication. In the context of the gut, this would be favourable as it guarantees persistence. It may also be that “Royal Family” ecological dynamics are at play which is the occurrence of “kill-the-winner” dynamics at a strain or sub-strain level, resulting phage-host fluctuations going undetected at genus or species level [57]. This is supported by the observed “host-jumping” of phages at the strain level which requires as little as a single point mutation in the tail fiber gene [58]. This phenomenon most likely occurs due to the inability of the phage to access the original host strain or as a result of a reduction in the cognate host strain counts due to variations in the gut environment. Interestingly, enrichment of non-synonymous mutations in the tail fibre gene of p-crAssphage has been observed with a greater incidence compared to other genes [39]. Strain level variation can be difficult to identify as 16S rRNA gene sequencing does not detect below species level [59]. Long-read sequencing, with platforms such as Oxford Nanopore, which can generate reads that are representative of near complete genomes may aid strain-level analyses [60, 61]. Bioinformatic pipelines that allow strain-resolved metagenomics have also been described [23, 62]. It is still unclear why *B. xylanisovlens* APCS1/XY seems to select for the presence of high titres of the phage while the bacterial count remains unaffected by its presence. Perhaps it is favourable to the host to prevent extinction of the phage. It may be that some ecological advantage is conveyed or that sub-strain level variation imposed by the presence of the phage is important in more complex situations than those examined in this study.

Phase variation is one possible mechanism that could allow hosts and phages to co-exist stably. This allows the host to transiently switch between phage permissive and non-permissive phenotypes through the reversible inversion of promotor-containing DNA regions called invertons at loci such as those encoding cell surface features which can act as phage receptors [30, 63]. Bacterial hosts have been shown to use phase variation to their advantage by developing herd immunity on phage exposure via phenotypic switch control of predating phage viral load [64]. This is possible due to the constant presence of a transiently phage permissive sub-population with the other portion of the population in a non-permissive state. This permits phage propagation but limits the viral load so that the host is never completely eliminated. A sub-population of each culture may revert to non-phage permissive at a certain threshold and thus control viral load. It has also been demonstrated in murine models that host permissiveness to phage infection is not uniform throughout the gut and is influenced by ecophysiology [65, 66]. Recently, the significance of transient resistance conveyed by phase variation and how it can dictate phage-host interactions has become of interest [30, 63, 64]. Examination of the *B. xylanisovlens* APCS1/XY genome revealed a large number of genes coding for outer membrane proteins and capsular polysaccharide biosynthesis enzymes, associated in many cases with potential invertons (GenBank CP042282, Additional file 7: Figure S4a-d) [30, 31, 67–69]. One study demonstrated that phase variable invertons are particularly dominant among human gut Bacteroidetes with ~19 invertons per genome [63]. Another noteworthy characteristic of phase variation detected in vitro is the inability of the phage to clear liquid culture despite reaching a high titre [30]. This is consistent with behaviour observed for both ΦcrAss002 and ΦcrAss001 [20]. Furthermore, this may explain the observed variability of opacity in zones of clearing and co-culture titres using cultures produced on different days and originating from different single colonies of *B. xylanisovlens* APCS1/XY.

In vivo studies with relevant conditions and transcriptomics could potentially expand our understanding of how *B. xylanisolvens* and ΦcrAss002 interact and co-exist. The persistence of virulent gut phages and their bacterial hosts was examined in a murine model using the defined Oligo-Mouse-Microbiota (OMM) bacterial consortium with the addition of two *E. coli* strains (murine commensal strain Mt1B1 and enteroaggregative strain 55989) with three virulent phages infecting Mt1B1 and one infecting 55989 [49, 70]. Interestingly, it was found that the radial variation in the murine gut due to anatomical features and condition gradients allows for variable virulent phage-host accessibility. Phage replication appears to largely occur in the lumen whereas the mucosa crypts provide a site of refuge for part of the target bacterial population which gradually migrate into the lumen. Further supporting this was the identification of an increasing mucosa to lumen gradient of lytic phages. Therefore, hosts are exposed to variable phage concentrations in the gut highlighting the significance of spatial heterogeneity [49]. This is also supported by observations of non-uniform phage propagation and composition throughout the GIT [65, 66, 71]. This work also suggests that arm-race dynamics and extension of host range do not have a role in persistence of virulent phages [49]. With strain variation among gut bacterial species often linked with genetic changes associated with phage resistance, the rate of and occurrence of genetic versus transient phage resistance requires further analysis [72]. Overall, the study specifically provides valuable insights into how persistent propagation of virulent phages can occur in the human gut without host elimination. Spatial separation in parallel with strain level interactions and transient host resistance may have important roles in this persistence.

## Conclusions

We report the isolation of ΦcrAss002 from the human gut following antibiotic driven enrichment of Bacteroidales in a faecal fermenter. Biological and in silico characterization of ΦcrAss002 revealed a number of interesting traits including the inability to form plaques or clear liquid cultures of its host despite the phage being lytic in nature and attaining high titres. Like ΦcrAss001, ΦcrAss002 can co-exist with its host over time without impacting host abundance at the species level. In the context of the gut, we hypothesize that multiple phenomena are occurring in parallel to allow such persistence and co-existence including “piggyback-the-winner” and “Royal Family” ecological dynamics, transient host phenotypic variations and spatial heterogeneity. The isolation of more crAss-like phages will be key to expanding our understanding of the most abundant phage family in the human gut. They infect one of the most abundant and important bacterial groups in the gut, Bacteroidales, and so these phages and their hosts provide an opportunity to study the dynamics involved in phage-bacterium interactions in microbial functionality within the gut. Understanding such interactions will be necessary if we are to comprehend the role of the phageome in bacterial homeostasis in the gastrointestinal tract.

## Methods

### Donor recruitment and sample collection

A healthy female donor in her forties, denoted as subject ID: 924, was recruited for faecal sample donation in October 2017. The individual was previously identified as being a persistent carrier of crAss-like phages over a period of 2 years [4, 19]. Therefore, this subject was deemed as a donor of interest for the isolation of potential novel crAss-like phages in vitro. Sample collection was in accordance with the study protocol APC055 and ethics approved by Cork Research Ethics Committee.

### Faecal fermentation

On receipt, the sample was processed into frozen standard inoculum (FSI). This was done as described by [73] with modifications. Faecal sample was resuspended in ×1 phosphate-buffered saline with 0.05% (w/v) L-cysteine (Sigma Aldrich, Ireland) and (1 mg/L) resazurin (Sigma Aldrich, Ireland). The crAssphage-rich FSI was inoculated into 400 ml YCFA-GSCM broth in a 500-ml fermenter vessel at 5% (v/v). Fermentation media was prepared as described in [19]. Triplicate fermentations were run in batch format over 24 h with conditions applied as by Guerin et al. [19]. Two fermenter vessels were set up in parallel, one with and one without the addition of antibiotics to the YCFA-GSCM broth post-autoclaving. Added antibiotics included 7.5 μg/ml vancomycin and 100 μg/ml kanamycin. The former was chosen based on its ability to suppress a broad range of Gram-positive bacteria [74] and the latter to limit faster growing facultative anaerobes. Dissolved oxygen was sustained at <0.1% by constantly sparging the vessel with anaerobic gas mix (80% (v/v) N2, 10% (v/v) CO_2_, 10% (v/v) H2) and stirring at 200 rpm. Both 2M NaOH and HCl solutions were used to maintain pH at ~7. Samples were collected at 0, 4.5, 17.5, 21 and 24 h and were directly processed after collection through centrifugation at 4700 rpm for 10 min at +4°C. Following this, supernatants were passed through a 0.45-μM pore polyethersulfone (PES) membrane filter and the resulting filtrates were stored at +4°C. The remaining bacterial rich pellets were stored at −80°C**.**

### Extraction of viral nucleic acids, virome library preparation and analysis

VLPs were enriched and viral nucleic acids extracted from 10 ml of filtered fermentation supernatants using the protocol as previously described [75]. Reverse transcription (RT) reaction was performed using SuperScript IV First-Strand Synthesis System (Invitrogen/ThermoFisher Scientific) with 11 μl of purified VLP nucleic acids sample and random hexamer oligonucleotides according to manufacturer’s protocol. One microlitre of the product was subjected to multiple displacement amplification (MDA) using Illustra GenomiPhi V2 kit (GE Healthcare) according to manufacturer’s instructions. This was performed in triplicate for each sample. MDA replicates were pooled together, combined with 7 μl of the remaining un-amplified RT product and purified using DNeasy Blood & Tissue kit (QIAGEN). Shotgun libraries were prepared using Nextera XT DNA Library Preparation Kit (Illumina). The shotgun libraries were then sequenced on an Illumina HiSeq 4000 platform with 2 × 150 nt paired-end chemistry at GATC Biotech AG, Germany.

The quality of the raw paired-end reads was analysed using FastQC v0.11.5. Trimming and filtering of the reads were performed with Trimmomatic v0.36 [76]. Parameters implemented were as follows: minimum length of 60, a sliding window size of 4 and a minimum Phread score of 33. The trimmed and filtered reads (~100M) were then assembled by sample into contigs using metaSPAdes v3.13.1 using standard parameters [77]. Contigs originating from all samples were pooled together and made non-redundant with a cut-off level of 90% sequence similarity over 90% of length of a shorter contig in each matched pair. Only longest representative contigs from each cluster of similar contigs were used to compile the final contig database (*n* = 6124; Additional file 10: Sequences of non-redundant contigs in FASTA format).

Contigs that correspond to crAss-like phages were then identified using BLASTn v2.2.28+ against a database of 249 crAss-like phages [19, 78] with *e* value cut-off level of ≤ 10^-10^, and a coverage cut-off > 90% of contig length at > 50% identity. A read count table by aligning quality-flitered and trimmed read against the database of non-redundant contigs using Bowtie2 v2.3.0 [79] in the ‘end-to-end’ mode. Samtools v0.1.19 commands ‘idxstat’ and ‘flagstat’ were used to extract aligned read counts. This was done to determine the relative abundance reads that resolve into crAss-like phage family genera (Additional file 11: Table S5). Virome diversity metrics for the antibiotic versus non-antibiotic containing vessels were calculated using R package Vegan v2.4.3

### Total DNA extraction and 16S rRNA gene sequencing library preparation

Total DNA was extracted from faecal pellets collected from centrifugation of fermentation samples. Extractions were performed using the QIAamp Fast Stool Mini Kit (Qiagen, Hilden, Germany). Approximately 200mg of each pellet was weighed into a 2-ml screw-cap tube containing a combination of glass beads varying in size—one 3.5mm glass bead, ~200 μl pf 1m zirconium beads and ~200 μl of 0.1 mm (Thistle Scientific). Proceeding extraction, PCR amplification of V3–V4 fragment of 16S rRNA gene, sequencing library preparation and data processing steps were performed as described by Shkoporov and colleagues [75]. Briefly, initial quality filtering was performed with Trimmomatic v0.36. The filtered reads were imported into R v3.4.3 and were analysed for errors using DADA2 package (v1.6.0) [80]. Identified errors were corrected via further quality filtering and trimming resulting in unique Ribosomal Variant Sequences (RSVs). The RSVs were subjected to chimera filtering using USEARCH v8.1 with the ChimeraSlayer gold database v20110519. The remaining RSVs were classified using RDP database v11.4 via the RDP-classifier in mothur v1.34.4 [81]. Species assignment was also performed using SPINGO [82]. The resultant RSVs were further analysed. This data was then used to determine the relative abundance of bacterial orders within the antibiotic and non-antibiotic containing vessels, which was visualised using the R package ggplot2 v2.2.1. The 16S diversity metrices for the antibiotic versus non-antibiotic containing vessels were also calculated using R Package Vegan v2.4.3.

### Absolute composition and quantitative real-time PCR

The absolute composition of detected crAss-like phage strains throughout the fermentations was determined using quantitative real-time PCR (qPCR) with the standard curve method. Primers were designed to target a consensus region of the terminase gene for each candidate genus detected in the fermenter. Where possible, genus-specific primers were designed based on terminase gene alignments as annotated by Guerin and colleagues [19]. Due to the heterogeneity of candidate genus VI and the genetic code variations observed for candidate genus VII crAss-like phages, primers were designed with species-level specificity. Primer sequences are listed in (Additional file 12: Table S6). PCR products generated from these primers were cloned into pCR2.1-TOPO TA vector (Thermo Fisher Scientific) to develop standards. Extracted plasmids were quantified using Qubit dsDNA BR Assay kit and diluted to 10^9^ copies/μl based on molar mass of DNA. Ten-fold serial dilutions of the plasmids were used to build a standard calibration curve. Threshold cycle (Ct) values demonstrated linear dependence (*R*^2^ = 0.99) from plasmid DNA concentration in the range from 10^1^ to 10^9^ copies/μl. The PCR efficiency was ~100%. Absolute quantification qPCR was performed with a 15-μl reaction volume using SensiFAST SYBR No-ROX mastermix (Bioline) in a LightCycler 480 thermocycler with the following conditions: initial denaturation at 95°C for 5 min, then 45 cycles of 95°C for 20 s, 60°C for 20 s and 72°C for 20 s. Resulting Ct values were converted to copies/ml based on the generated calibration curves. Results were visualised using GraphPad Prism v8.0 software.

### Screening for novel crAss-like phages from faecal fermentates

Bacteroidales were enriched for from the FSI preparation. Ten-fold serial dilutions of the FSI were prepared in fresh Fastidious Anaerobe Broth (FAB, Neogen), and 100 μl of each was spread plated on Fastidious Anaerobe Agar (FAA, Neogen), YCFA-Agar, and Columbia Blood Agar (Oxoid) with 5% sheep blood supplemented with 25 μg/ml haemin and 100 μg/ml vitamin K. To each of these media, 7.5 μg/ml vancomycin and 100 μg/ml kanamycin was added post autoclaving. The dilution plates were anaerobically incubated at 37°C for 48 h, and formed colonies were restreaked. Approximate species identification was performed by Sanger sequencing of the 16S rRNA region using the universal bacterial primers 1492R 5′-GGTTACCTTGTTACGACTT-3′′ [83] and Bact 8 F 5′-AGAGTTTGATCCTGGCTCAG-3′ [84]. Samples were prepared as per the instruction for LightRun Tube service (GATC) analysed via online BLASTn (standard parameters) against the NCBI 16S ribosomal RNA sequences (Bacteria and Archaea) database.

Phage-bacterium host pair screening was performed in biological triplicate by co-culturing. Overnight cultures were prepared from the purified Bacteroidales strains. Ten microlitres was sub-cultured into 400 μl of fresh FAB, with cofactors MgSO_4_ and CaCl_2_ at a final concentration of 1 mM, contained within deep well plates (Sigma-Aldrich). The cultures were incubated anaerobically at 37°C until early logarithmic phase of growth. An OD_600_ = ~0.2 was measured approximately 5 h post sub-culturing. To the early log phase cultures, 100 μl of crAss-like phage rich fermentate filtered of bacterial cells was added and incubated anaerobically at 37°C for 24 h. Without centrifugation or filtering, 10 μl of the phage-bacteria mix was directly sub-cultured (1:50) into fresh FAB. Sub-culturing was repeated over three consecutive days. Total DNA was extracted using DNeasy Blood & Tissue Kit (Qiagen) and analysed for phage propagation on a specific host by qPCR analysis (Additional file 12: Table S6).

The detected phage-host pair, ΦcrAss002 and *B. xylanisolvens* APCS1/XY, was enriched using the above co-culturing method in 10 ml volumes. Following five rounds of enrichment, viral nucleic acids were extracted using the protocol exactly as described by [20]. Library preparation for shotgun sequences was performed using the Accel-NGS 1S Plus DNA Library Kit (Swift Biosciences) according to the manufacturer’s protocol. After index PCR an additional bead clean-up was performed using a ratio of 1:1 DNA/AMPure beads. Sequencing was performed using a 2 × 150 nt paired end run on an Illumina HiSeq 4000 platform at GATC Biotech AG, Germany.

### Transmission electron microscopy

Ultra-centrifugation was performed using a 60-ml volume of ΦcrAss002 filtrate. The supernatant was concentrated for a total of 4 h at 120,000g using a F65L-6×13.5 rotor (Thermo Scientific). The resulting pellets were resuspended in a final volume of 5ml SM buffer. The suspensions were then applied onto to a step gradient of 5M and 3M CsCl solutions, followed by centrifugation at 105,000*g* for 2.5 h at +4°C. The CsCl clean-up steps following this were previously described [19].

Five microlitre aliquots of the concentrated viral fraction were applied to Formvar/Carbon 200 Mesh, Cu grids (Electron Microscopy Sciences), with subsequent removal of excess sample by blotting. Grids were then negatively contrasted with 0.5% (w/v) uranyl acetate and examined at UCD Conway Imaging Core Facility (University College Dublin, Ireland) by Tecnai G2 12 BioTWIN transmission electron microscope.

### Shotgun sequencing of *B. xylanisolvens* APCS1/XY using Illumina and Oxford Nanopore platforms

Genomic DNA was extracted from 10 ml of *B. xylanisolvens* APCS1/XY overnight culture using phenol/chloroform extraction with precipitation in 3M sodium acetate and cold absolute ethanol. Cultures were centrifuged at 5000*g* for 10 min, and pellets were resuspended in 1 ml deionised water. The protocol was then performed as described by Sambrook et al. with the modifications implemented by Bardina et al. [85, 86]. Following precipitation, the DNA was resuspended in 50 μl Tris-EDTA buffer and incubated at 37°C to aid resuspension.

A long read Oxford Nanopore library preparation was performed as described per the user manual for Rapid Barcoding Sequencing Kit (SQK-RPK004; Oxford Nanopore Technologies) with the following modifications: 800ng of each sample was used and final pellet resuspension was carried out using 20μl of nuclease-free water pre-warmed to 65°C followed by a 10-min incubation at room temperature. Pooled samples were loaded into SpotON Flow Cell (Oxford Nanopore Technologies) and MinION sequenced for 48 h (Oxford Nanopore Technologies). Short-read shotgun sequencing of the extracted DNA was performed as described above using Accel-NGS 1S Plus DNA Library Kit and Illumina HiSeq 4000 platform. Hybrid assembly of quality-filtered and trimmed Illumina and raw Nanopore reads was performed using SPAdes (v1.13.1) [87, 88] to generate 3 circular scaffolds with a sequencing depth of 43.0×, corresponding to *B. xylanisolvens* APCS1/XY chromosome (6.4 Mbp) and two plasmids (5.6 and 4.1 kbp). The assembled and circularised scaffolds were annotated using to NCBI Prokaryotic Genome Annotation Pipeline (PGAP). The GenBank file for the genome was visualised using GView (v1.7) [89].

Dynamic local recombination hotspots in the *B. xylanisolvens* APCS1/XY chromosome were detected by aligning Oxford Nanopore reads to the assembled scaffolds. Alignment was performed using BLASTn, and only reads with length >1000 nt were considered (*n* = 155,425). Individual local alignments of >90% identity (*e* value <1e−20) and >200nt length were kept. All internal inversions or shifts in alignment coordinates versus reference genomic scaffold >200 nt were deemed as recombinations. Recombination hotspots were identified when >8 reads with inconsistent alignment were present per 1000bp window of genome length.

### In silico characterisation of ΦcrAss002

Annotation of ΦcrAss002 genome was performed using the de novo viral genome annotator VIGA [90]. The predicted protein coding sequences were further analysed with HHPred using the following databases: PDB_mm_CIF70_28_Dec, Pfam-A_v31.0, NCBI_CD_v3.16, TIGRFAMs_v15.0 [91]. A genomic map of the ΦcrAss002 genome was then generated using GView (v1.7). To examine the DNA termini and packing mechanism employed by ΦcrAss002, the command line version of PhageTerm v1.0.12 was used [92].

The ΦcrAss002 genome was examined against other crAss-like phages of candidate genus IV using BLASTn to examine relatedness [19]. Average nucleotide identity (ANI) was determined using the default settings in PYANI [93]. The output was exported into R environment to generate a heatmap with Bioconducter Complex Heatmap (v1.20.0) and Circlize (v0.4.5) packages [94, 95]. A pairwise comparison of the genomes was performed to examine synteny using tBLASTx in Easyfig (v2.2.2) with a minimum alignment length of 50bp and 30% identity. Genome comparison were also performed to investigate the synteny of ΦcrAss002 in comparison to ΦcrAss001 (GenBank MH675552.1) and p-crAssphage (GenBank NC_024711.1). Conserved protein sequences (major capsid protein and large terminase subunit) were aligned using MUSCLE v3.8.31 and approximately-maximum-likelihood phylogenetic trees were generated using FastTree v2.1.10 with default parameters.

### Biological characterisation of ΦcrAss002

Plaque assays were performed using 10-fold serial dilutions of ΦcrAss002 lysate prepared in SM buffer with an overlay of 0.3% FAA agar (0.3% agar w/v), containing MgSO_4_ and CaCl_2_ at a final concentration of 1 mM. To 3 ml of molten overlay, 300 μl *B. xylanisolvens* APCS1/XY overnight culture was added and 50 μl of phage dilution. This mixture was vortexed and poured onto pre-prepared FAA base agar (1.5% agar w/v). Plates were incubated anaerobically at 37°C. Plaque formation was checked at 24 and 48 h. Spot assays were performed as described for plaque assays but without addition of phage to the molten overlay agar. A 10-μl drop of phage was directly applied to the solidified lawn of *B. xylanisolvens* APCS1/XY and dried prior to incubation.

Attempts to generate a one-step growth curve were performed by infecting an early logarithmic phase culture of *B. xylanisolvens* APCS1/XY with ΦcrAss002 at a multiplicity of infection (MOI) of 1. Following incubation at room temperature for 5 mins, centrifugation was performed at 5000 rpm in a swing bucket rotor for 15 min at 20°C. The supernatant was removed, and the resultant pellet was resuspended with FAB. Anaerobic conditions were maintained at 37°C for 3 h with 1 ml sample collection every 15 min. Samples were centrifuged and filtered through 0.45-μM pore syringe filters. Analysis was performed using qPCR as described above using CGIV_Fwd and CGIV_Rev primers (Additional file 12: Table S6).

The ability of ΦcrAss002 to infect another commercially available *B. xylanisolvens* strain, DSM 18836 (DSMZ), and the ΦcrAss001 host, *B. intestinalis* 919/174, was examined by co-culture and the standard propagation method of centrifuging and filtering phage lysate between propagations and re-exposure to the host. Efficiency of lysogeny was performed using 100 μl of ΦcrAss002 at ~10^9^ pfu/ml spread plated on FAA agar plates. One hundred microlitres of 10-fold serially diluted *B. xylanisolvens* APCS1/XY overnight culture was added to 3ml of molten 0.3% FAA soft agar with co-factors and was poured onto the phage seeded plates. Negative controls were prepared in the same manner but without the addition of phage to the plate. The plates were incubated anaerobically at 37°C for 48 h. Efficiency of lysogeny was calculated as the percentage of colonies on the phage seeded plate versus counts for the equivalent negative control. Thirty resistant colonies were restreaked three times. Standard PCR was performed using ΦcrAss002-specific primers to test for potential lysogens.

### Co-cultivation of ΦcrAss002 and *Bacteroides xylanisolvens* APCS1/XY

Co-cultivation of ΦcrAss002 and *B. xylanisolvens* APCS1/XY was performed to examine propagation dynamics over time in vitro via serial sub-culturing of phage and host. This was initiated using 10 ml of culture (OD_600_ = ~0.2) prepared from naïve *B. xylanisolvens* cells i.e. ~10 generations without exposure to ΦcrAss002 and 1ml of phage lysate at ~10^9^ pfu/ml. Subsequent rounds of sub-culturing were performed by introducing the prior co-culture into fresh FAB at a ratio of 1:50. This was repeated over 11 days, and ΦcrAss002 titre quantification was performed via qPCR. The phage lysate generated from the final time point was then used to initiate another round of serial co-cultivation over 30 days. Having observed a propagation pattern, a third round of experimentation was initiated over 6 days. On day 6, the co-culture was centrifuged for 15 min at 5000 rpm in a swing bucket rotor at 4°C. The supernatant was filtered through 0.45-μM pore syringe filters. The resultant pellet was retained and t-streaked for three generations to purify the bacteria of phage. The absence of adsorbed or integrated ΦcrAss002 was confirmed via qPCR. Ten *B. xylanisolvens* colonies, that were recently exposed to ΦcrAss002 but were free of any remaining phage, were used to initiate a final cycle of co-cultivation. Absolute quantification of ΦcrAss002 titre was performed via qPCR (Additional file 12: Table S6). Significant differences between the ΦcrAss002 titres sustained by the cultures were evaluated using one-way ANOVA followed by Tukey’s post hoc comparisons. Spot assays of ΦcrAss002 were performed with each of the clones to test for resistance or changes in spot opacity.

### Examination of ΦcrAss001 and ΦcrAss002 dynamics in a fermenter system with a defined bacterial consortium

An unusual phage-host equilibrium has been observed for both of the crAss-like phages isolated to date. This was further examined in a fermenter system with a defined community of commensal bacteria. This defined community was compiled of six bacteria in addition to *B. xylanisolvens* APCS1/XY and *B. intestinalis* 919/174: *E. coli* LF82, *E. faecalis* OG1RF, *Ruminococcus gnavus* ATCC 29149, *Faecalibacterium prausnitzii* A2-165, *Lactobacillus plantarum* WCFS1, and *Bifidobacterium longum* subsp. *longum* ATCC 15707. Collectively, these human gut derived bacteria are referred to as a simplified human consortium (SIHUMI) [96]. Our community excluded *Bacteroides vulgatus* ATCC 8482 to avoid issues with discrimination between the *Bacteroides* phage hosts.

Fermentations were performed in batch format for the first 24 h to allow establishment of the bacterial community. Overnight cultures were grown to ~10^9^ cfu/ml and combined at a ratio of 1:1. The fermenter vessels, containing 200 ml of YCFA-GSCM broth, were then inoculated with 1ml of this mixture. After 24 h, 10 ml of ΦcrAss001 (~10^9^ pfu/ml) and 10 ml of ΦcrAss002 (~10^8^ pfu/ml) were inoculated into one vessel. The other remained without phage in parallel to act as a control. For 4 days, a continuous fermentation was performed with 400 ml of media exchanged in every 24 h with equivalent waste removal. Samples were collected at the following time points: 0, 6, 24, 48 and 72 h post-phage inoculation and were frozen directly at −80^o^C. Fermentation runs were performed in triplicate using aliquots from the same phage lysate. Total DNA was extracted using the QIAamp Fast Stool Mini Kit (Qiagen, Hilden, Germany). All DNA samples were normalised prior to analyses by diluting to 5 ng/μl. Bacterial primers were designed targeting unique genes (Additional file 13: Table S7.) and were used to develop qPCR standards with the method described previously. Primers specific to the portal protein were used in ФcrAss001 qPCR analyses [20]. Conditions for qPCR were as follows: initial denaturation at 95°C for 5 min, then 45 cycles of 95°C for 20 s, 62°C for 20 s and 72°C for 20 s. For bacterial analyses an annealing temperature of 62°C was used and when analysing both phages, 60°C. Results were visualised using GraphPad Prism v8.0 software.

## Supplementary Information

**Additional file 1: Figure S1.** Graphical representation of the key experimental steps taken in the isolation of ΦcrAss002.

**Additional file 2: Figure S2.** Diversity index of fermentates generated with and without selective conditions. a 16S diversity index. In the presence of antibiotics alpha-diversity, evenness and richness are decreased. b Virome diversity index. Under selective enrichment there is a reduction for each index in parallel with bacteriome reduction. Error bars indicate standard deviation between triplicate fermentations (*n* = 3).

**Additional file 3: Table S1.** Bacterial isolates identified following Sanger sequencing. The isolates were enriched from subject ID: 924 faeces with the aid of antibiotic selective enrichment to promote Bacteroidales growth. FAA; Fastidious anaerobic agar, YCFA; yeast extract, casitone, fatty acids agar, CBA; Columbia blood agar.

**Additional file 4: Table S2.** Functional annotation of ΦcrAss002 protein-coding genes using HHpred.

**Additional file 5: Table S3.** Metadata associated with ΦcrAss002 and related phages of candidate genus IV identified by in silico analyses.

**Additional file 6: Figure S3.** Phylogenetic tree of large terminase subunits encoded by published complete genomes of crAss-like phages. Protein sequences were aligned using MUSCLE, approximately-maximum-likelihood phylogenetic trees were generated using FastTree. Branch support values calculated using SH-test. Tree tip colours correspond to candidate genera as proposed in [19]. ΦcrAss002 label is highlighted in red. Previously well-characterised uncultured phage genomes and cultured isolates are highlighted in boldface font. Accession numbers of genomes in NCBI GenBank/RefSeq/WGS databases are provided.

**Additional file 7: Figure S4.** Multiple site-specific recombinase-encoding genes and evidence of dynamic recombinations in the genome of *B. xylanisolvens* APCS1/XY. a Circular map of genome (the innermost circle [green and purple], GC skew; circle two [black], relative G+C content; circles three and four [red and dark blue], open reading frames identified on the positive and negative DNA strands respectively; circle five [orange], tRNA and rRNA genes. circle six, genes annotated as Sus-like surface-associated glycan utilisation proteins [green] and TonB-dependent nutrient receptor [light blue]; circle seven [black], genes annotated as invertases, integrases and recombinases). To the right of the genome map, circular maps of the two associated circular plasmids are shown; pBXS1-1 and pBXS1-2. Annotated features are coloured and labelled; b Distribution of length of Oxford Nanopore sequencing reads used for dynamic genome recombination analysis; c Distribution of percentage identity in Oxford Nanopore reads aligned using BLASTn to the chromosome scaffold; d Frequency of detected recombinations at a single read level (reads of at least 1000nt, with individual alignments of >90% identity and >200nt length; all inversions or shifts in coordinates >200 nt were deemed as recombinations) versus coordinates in the chromosome scaffold (histogram bin size = 1000bp); recombination hotspots were identified when >8 reads with inconsistent alignment were present per 1000bp bin; gene products overlapping with hotspots are marked on the plot.

**Additional file 8: Figure S5.** Transmission electron micrographs showing vesicle-like structures on the surface of *B. xylanisolvens* APCS1/XY cells. Micrographs were prepared from cross-sections of soft agar collected lawns of *B. xylanisolvens* APCS1/XY with and without spotting of ΦcrAss002 lysates.

**Additional file 9: Table S4.** Summary of ΦcrAss001 and ΦcrAss002 characteristics.

**Additional file 10:** Sequences of non-redundant contigs in FASTA format.

**Additional file 11: Table S5.** Partial and complete genomic contigs of crAss-like phages. (XLS 9 kb)

**Additional file 12: Table S6.** Primer sequences specific to each crAss-like phage strains detected following sequencing of subject ID: 924 faeces post fermentation.

**Additional file 13: Table S7.** Primers specific to the SIHUMI bacterial community, ΦcrAss001 host B. intestinalis 919/174, and ΦcrAss002 host B. xylanisolvens APCS1/XY.

## Data Availability

Raw metagenomic sequencing data (VLP shotgun sequencing and 16S rRNA amplicon sequencing) from faecal fermentation experiment is available under NCBI BioProject PRJNA701013. The genome of ΦcrAss002 is deposited into GenBank under accession MN917146 (assembled and annotated genome). The genome of the bacterial host of ΦcrAss002, *Bacteroides xylanisolvens* APCS1/XY, is deposited under the following accession codes: BioProject PRJNA556867, GenBank CP042282 (assembled and annotated genome) and associated plasmids pBXS1-1 and pBXS1-2, GenBank CP042281 and GenBank CP042283, respectively.

## Abbreviations

ANI: Average nucleotide identity
CBA: Columbia blood agar
FAA: Fastidious anaerobe agar
FSI: Frozen standard inoculum
Host: Refers exclusively to the bacterial host unless stated otherwise
ICTV: International Capitalise Committee, Taxonomy, Viruses
MDA: Multiple displacement amplification
MOI: Multiplicity of infection
OMM: Oligo-mouse-microbiota
OMV: Outer membrane vesicles
PEG: Polyethylene glycol
PES: Polyethersulfone
PPV: Personal persistent virome
qPCR: Quantitative real-time PCR
SIHUMI: Simplified human consortium
VLP: Virus-like particle
VMR: Virus-to-microbe ratio
WGA: Whole-genome amplification
YCFA-GSCM: Yeast, casitone and fatty acids modified with glucose, sucrose, cellulose and maltose
