# Supplementary figures and images for: Isolation and characterisation of ΦcrAss002, a crAss-like phage from the human gut that infects Bacteroides xylanisolvens
Source: Microbiome. 2021 Apr 12;9:89. doi: 10.1186/s40168-021-01036-7 (PMC8042965; doi:10.1186/s40168-021-01036-7)

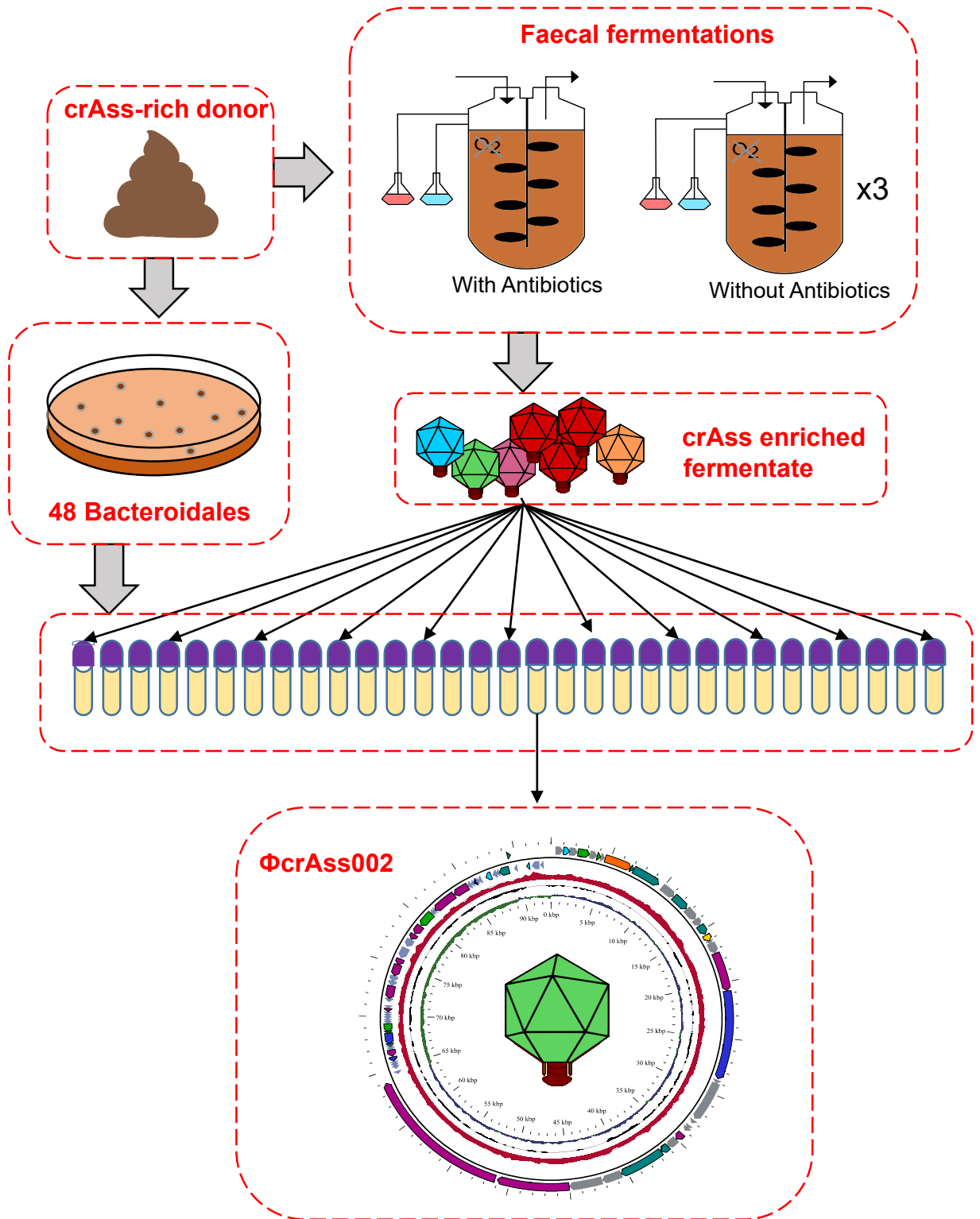

Supplement: Supplementary file 2 — Additional file 1: Figure S1. Graphical representation of the key experimental steps taken in the isolation of ΦcrAss002. [file 40168_2021_1036_MOESM2_ESM.pdf]

**a**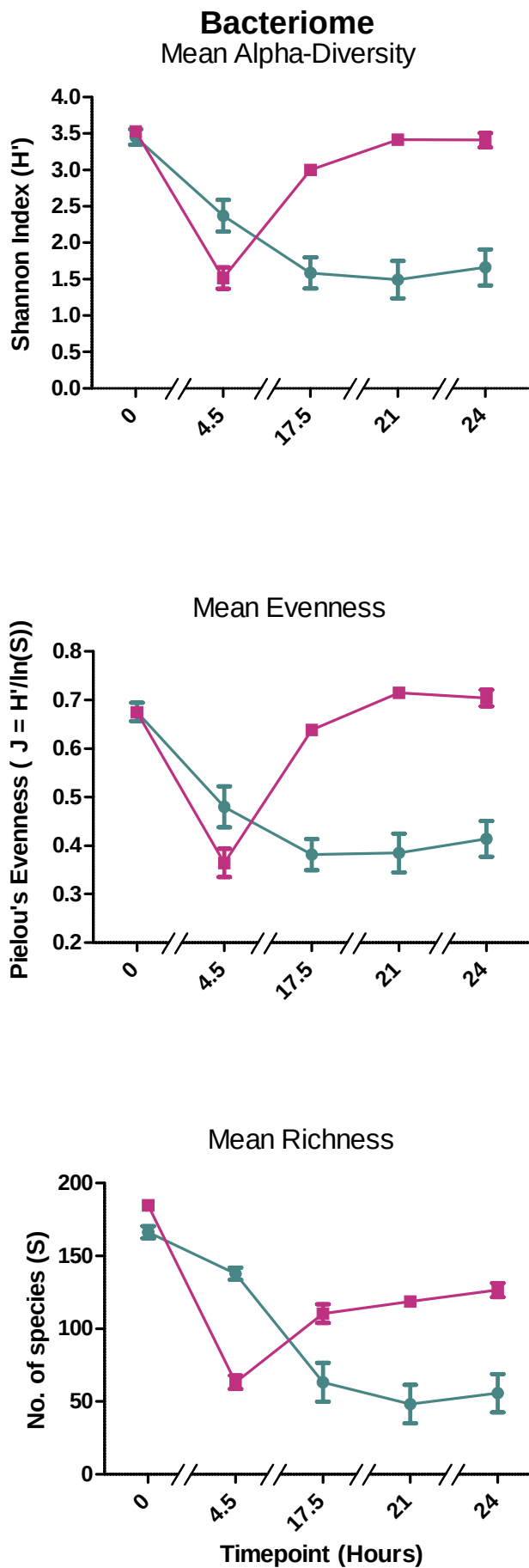**b**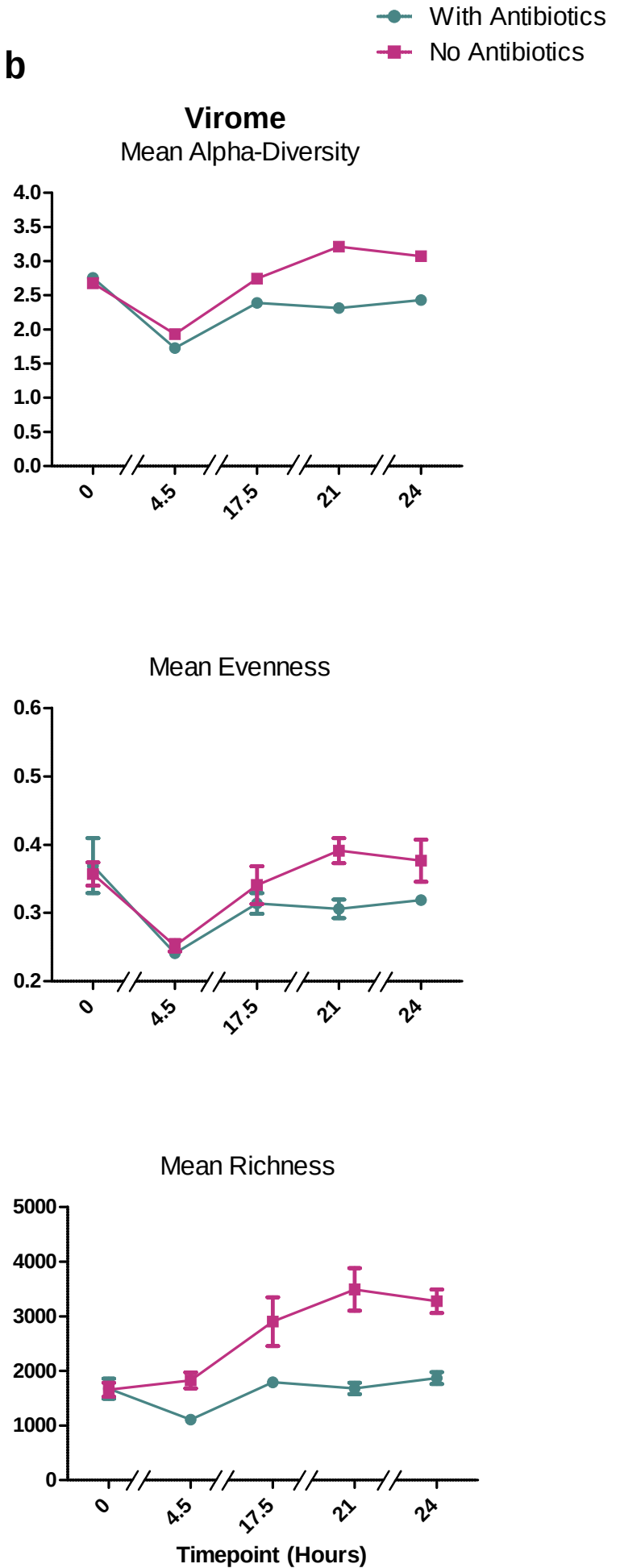

Supplement: Supplementary file 3 — Additional file 2: Figure S2. Diversity index of fermentates generated with and without selective conditions. a 16S diversity index. In the presence of antibiotics alpha-diversity, evenness and richness are decreased. b Virome diversity index. Under selective enrichment there is a reduction for each index in parallel with bacteriome reduction. Error bars indicate standard deviation between triplicate fermentations (n = 3). [file 40168_2021_1036_MOESM3_ESM.pdf]

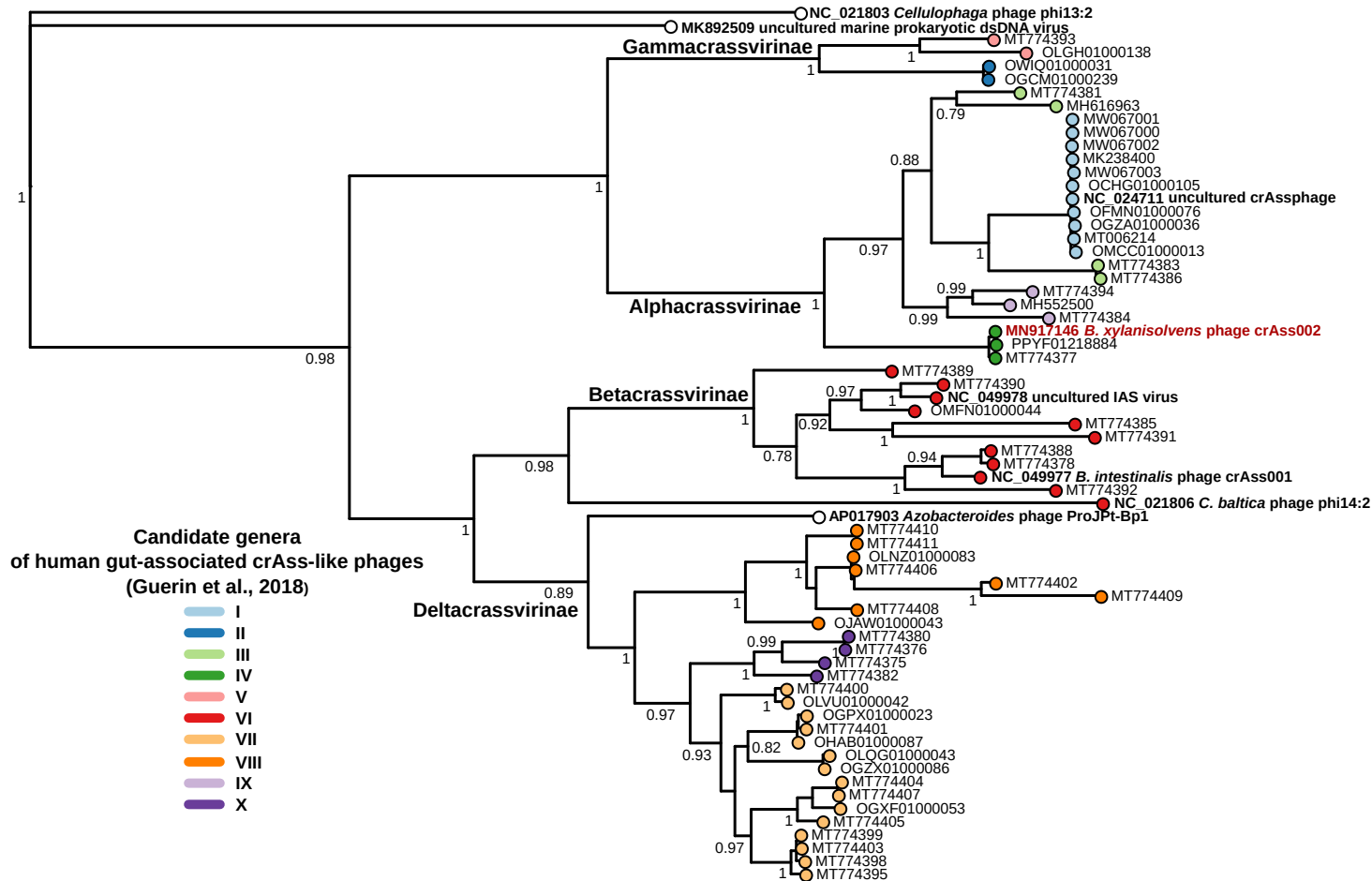

Supplement: Supplementary file 7 — Additional file 6: Figure S3. Phylogenetic tree of large terminase subunits encoded by published complete genomes of crAss-like phages. Protein sequences were aligned using MUSCLE, approximately-maximum-likelihood phylogenetic trees were generated using FastTree. Branch support values calculated using SH-test. Tree tip colours correspond to candidate genera as proposed in [19]. ΦcrAss002 label is highlighted in red. Previously well-characterised uncultured phage genomes and cultured isolates are highlighted in boldface font. Accession numbers of genomes in NCBI GenBank/RefSeq/WGS databases are provided. [file 40168_2021_1036_MOESM7_ESM.pdf]

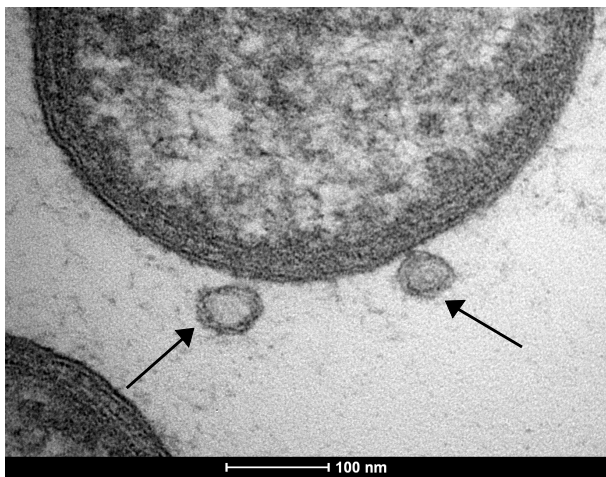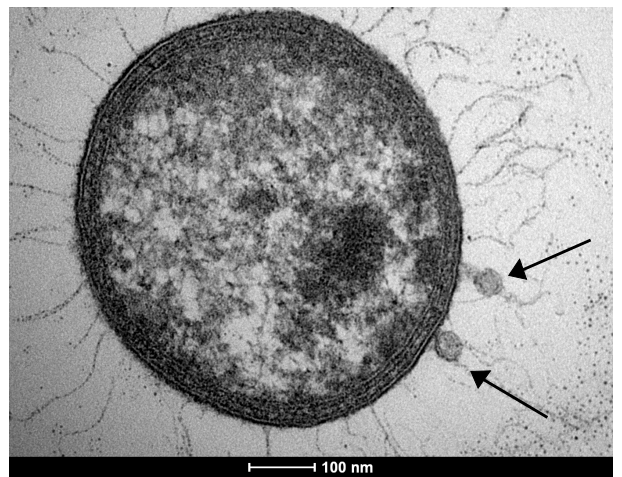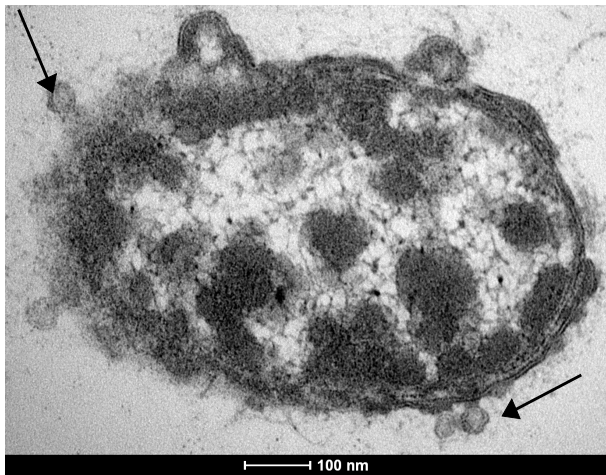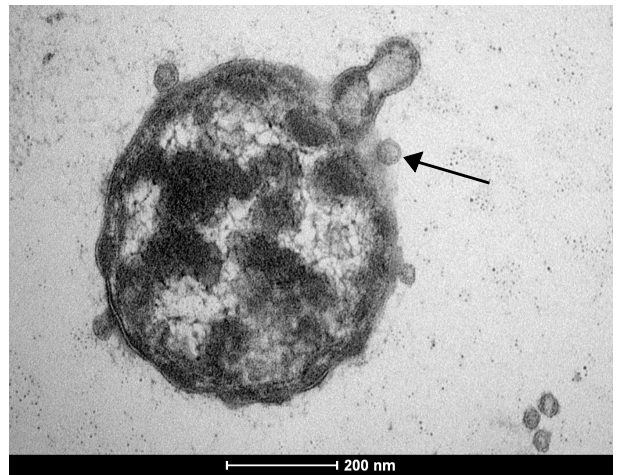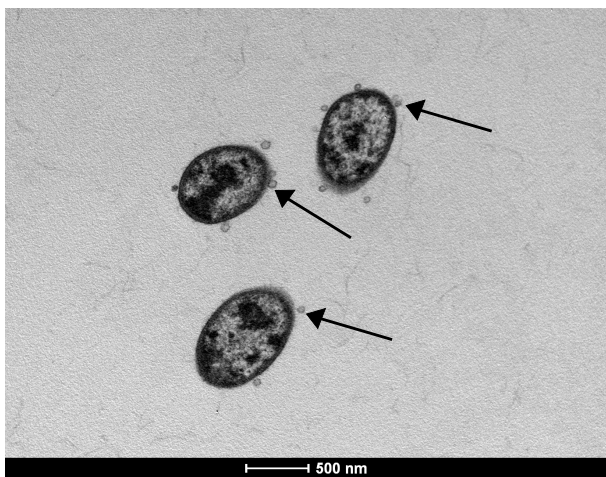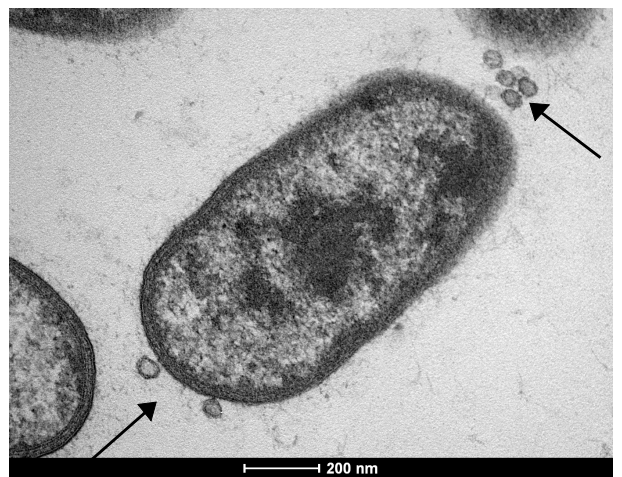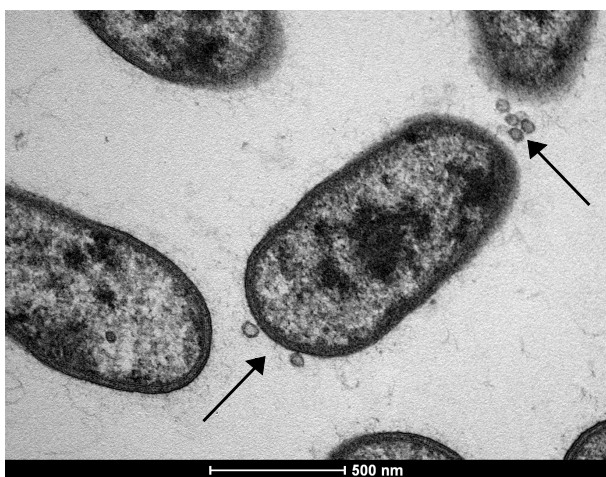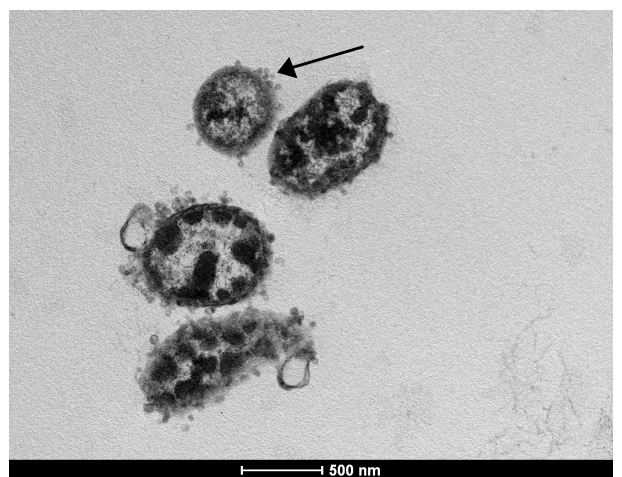

Supplement: Supplementary file 9 — Additional file 8: Figure S5. Transmission electron micrographs showing vesicle-like structures on the surface of B. xylanisolvens APCS1/XY cells. Micrographs were prepared from cross-sections of soft agar collected lawns of B. xylanisolvens APCS1/XY with and without spotting of ΦcrAss002 lysates. [file 40168_2021_1036_MOESM9_ESM.pdf]
