# Supplementary material for: Isolation and characterisation of ΦcrAss002, a crAss-like phage from the human gut that infects Bacteroides xylanisolvens
Source: Microbiome. 2021 Apr 12;9:89. doi: 10.1186/s40168-021-01036-7 (PMC8042965; doi:10.1186/s40168-021-01036-7)
Supplement: Supplementary file 8 — Additional file 7: Figure S4. Multiple site-specific recombinase-encoding genes and evidence of dynamic recombinations in the genome of B. xylanisolvens APCS1/XY. a Circular map of genome (the innermost circle [green and purple], GC skew; circle two [black], relative G+C content; circles three and four [red and dark blue], open reading frames identified on the positive and negative DNA strands respectively; circle five [orange], tRNA and rRNA genes. circle six, genes annotated as Sus-like surface-associated glycan utilisation proteins [green] and TonB-dependent nutrient receptor [light blue]; circle seven [black], genes annotated as invertases, integrases and recombinases). To the right of the genome map, circular maps of the two associated circular plasmids are shown; pBXS1-1 and pBXS1-2. Annotated features are coloured and labelled; b Distribution of length of Oxford Nanopore sequencing reads used for dynamic genome recombination analysis; c Distribution of percentage identity in Oxford Nanopore reads aligned using BLASTn to the chromosome scaffold; d Frequency of detected recombinations at a single read level (reads of at least 1000nt, with individual alignments of >90% identity and >200nt length; all inversions or shifts in coordinates >200 nt were deemed as recombinations) versus coordinates in the chromosome scaffold (histogram bin size = 1000bp); recombination hotspots were identified when >8 reads with inconsistent alignment were present per 1000bp bin; gene products overlapping with hotspots are marked on the plot. [file 40168_2021_1036_MOESM8_ESM.pdf]

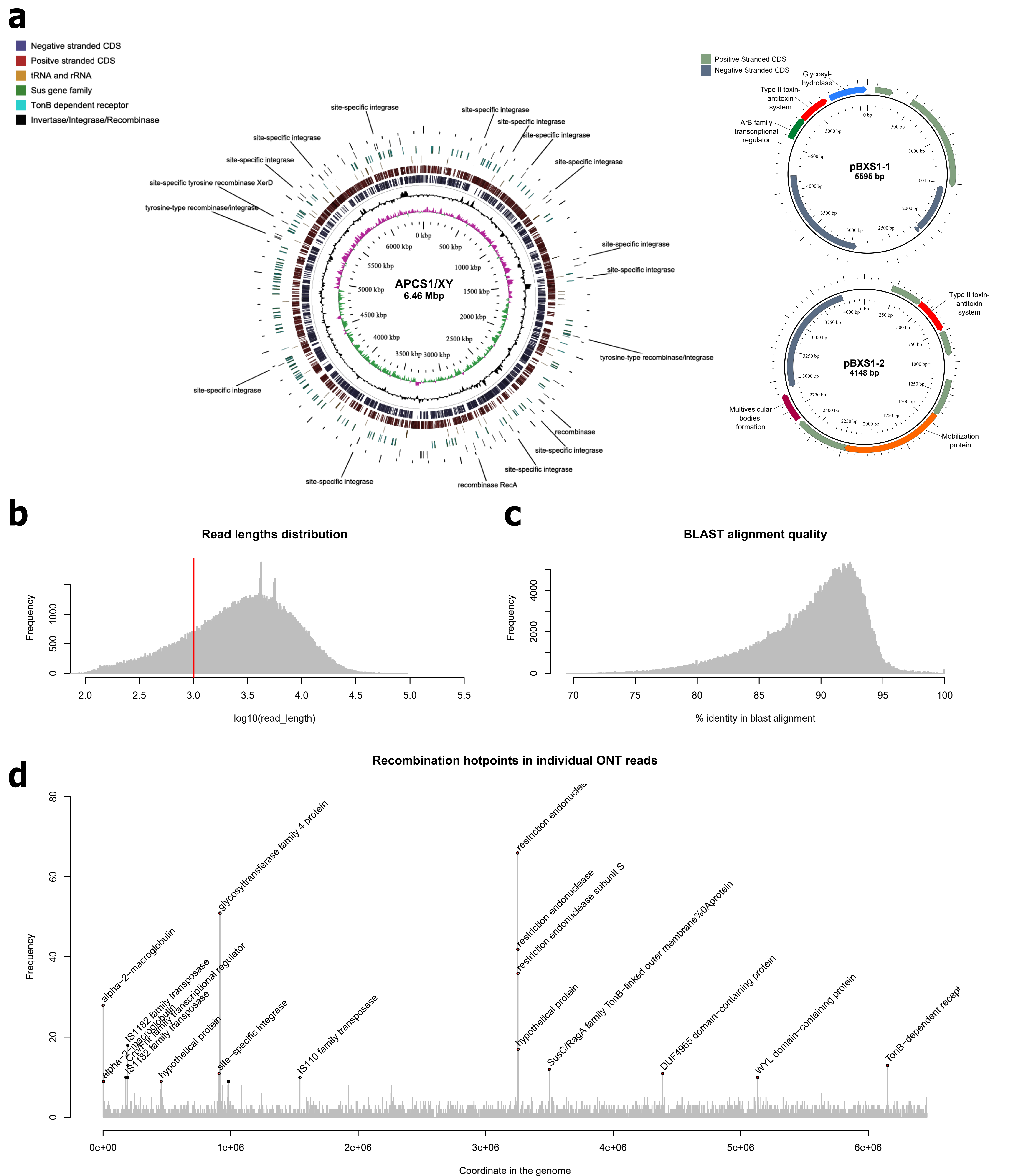
a
Negative stranded CDS
Positve stranded CDS
tRNA and rRNA
Sus gene family
TonB dependent receptor
Invertase/Integrase/Recombinase
site-specific integrase
site-specific tyrosine recombinase XerD
tyrosine-type recombinase/integrase
recombinase
recombinase RecA
APCS1/XY
6.46 Mbp
0 kbp
500 kbp
1000 kbp
1500 kbp
2000 kbp
2500 kbp
3000 kbp
3500 kbp
4000 kbp
4500 kbp
5000 kbp
5500 kbp
6000 kbp
Positive Stranded CDS
Negative Stranded CDS
Glycosyl-hydrolase
Type II toxin-antitoxin system
ArB family transcriptional regulator
pBXS1-1
5595 bp
pBXS1-2
4148 bp
Multivesicular bodies formation
Mobilization protein
b
Read lengths distribution
Frequency
log10(read_length)
c
BLAST alignment quality
Frequency
% identity in blast alignment
d
Recombination hotpoints in individual ONT reads
Frequency
Coordinate in the genome
alpha−2−macroglobulin
IS1182 family transposase
Crp/Fnr family transcriptional regulator
hypothetical protein
glycosyltransferase family 4 protein
site−specific integrase
IS110 family transposase
restriction endonuclease subunit S
restriction endonuclease
SusC/RagA family TonB−linked outer membrane%0Aprotein
DUF4965 domain−containing protein
WYL domain−containing protein
TonB−dependent recept
